# Efficacy and Safety of Oral Anticoagulants for Atrial Fibrillation Patients With Chronic Kidney Disease: A Systematic Review and Meta-Analysis

**DOI:** 10.3389/fcvm.2022.885548

**Published:** 2022-06-10

**Authors:** Tae-Min Rhee, So-Ryoung Lee, Eue-Keun Choi, Seil Oh, Gregory Y. H. Lip

**Affiliations:** ^1^Department of Internal Medicine, Seoul National University Hospital, Seoul, South Korea; ^2^Department of Internal Medicine, Seoul National University College of Medicine, Seoul, South Korea; ^3^Liverpool Centre for Cardiovascular Science, Liverpool Chest and Heart Hospital, University of Liverpool, Liverpool, United Kingdom; ^4^Department of Clinical Medicine, Aalborg University, Aalborg, Denmark

**Keywords:** atrial fibrillation, anticoagulation, chronic kidney disease, meta-analysis, direct oral anticoagulant

## Abstract

**Background:**

Data on different direct oral anticoagulants (DOACs) in atrial fibrillation (AF) patients with renal impairment are insufficient. We aimed to perform pairwise and network meta-analysis comparing oral anticoagulants (OACs) in AF patients with renal impairment, including advanced chronic kidney disease (CKD) with creatinine clearance <30 mL/min.

**Methods:**

PubMed, Embase, Cochrane Database, and references of related articles were searched up to April 2021. We included randomized trials and non-randomized studies using propensity-score or multivariable-model adjustments that compared clinical outcomes among OACs. Hazard ratios (HRs) for stroke or thromboembolism, major bleeding, and all-cause death were pooled using random-effects model.

**Results:**

From 19 studies, 124,628 patients were included. In patients with AF and CKD, DOACs presented significantly lower risks of stroke or thromboembolism [HR_pooled_ = 0.78, 95% confidence interval (CI) = 0.73–0.85, I^2^ = 16.6%] and major bleeding [HR_pooled_ = 0.76 (0.64–0.89), I^2^ = 85.7%] when compared with warfarin, regardless of the severity of renal impairment. Results were consistent in advanced CKD patients for stroke or thromboembolism [HR_pooled_ = 0.60 (0.43–0.85), I^2^ = 0.0%] and major bleeding [HR_pooled_ = 0.74 (0.59–0.93), I^2^ = 30.4%]. In the network meta-analysis, edoxaban and apixaban presented the highest rank probability to reduce the risk of stroke or thromboembolism (edoxaban, P-score = 94.5%) and major bleeding (apixaban, P-score = 95.8%), respectively. Apixaban remained the safest OAC with the highest rank probability for major bleeding (P-score = 96.9%) in patients with advanced CKD.

**Conclusion:**

DOACs, particularly apixaban and edoxaban, presented superior efficacy and safety than warfarin in AF patients with CKD. Apixaban was associated with the lowest risk of major bleeding among OACs for patients with advanced CKD.

**Systematic Review Registration:**

[PROSPERO], identifier [CRD42021241718].

## Introduction

The presence of chronic kidney disease (CKD) increases both thromboembolic and bleeding risks in patients with atrial fibrillation (AF) (1–3), which makes anticoagulation therapy challenging in this patient group (3). Although the introduction of direct oral anticoagulants (DOACs) has led to safer oral anticoagulation (OAC) therapy in general (4, 5), there are areas of uncertainty in patients with AF and CKD. Notably, patients with advanced CKD [creatinine clearance (CrCl) <30 mL/min] have been excluded from the pivotal randomized controlled trials (RCTs), except for some patients on apixaban with CrCl of 25–30 mL/min (6–10). In addition, few studies have directly compared DOACs in patients with CKD (11, 12).

After publication of the practical guidelines on DOAC use in patients with CKD provided by the European Heart Rhythm Association (13), several observational studies have been published the comparing various OACs in patients with CKD (14–27). We thus aimed to evaluate the pooled efficacy and safety of DOACs compared with warfarin in AF patients with various stages of CKD, including advanced CKD with CrCl <30 mL/min. Second, we performed a network meta-analysis to comprehensively evaluate and rank different OAC strategies, including type of DOAC and warfarin, in patients with AF and CKD.

## Materials and Methods

A detailed description of the study methods is presented in the Supplementary Materials.

### Data Sources and Search Strategies

We performed electronic searches of PubMed, Embase, Cochrane Central Register of Controlled Trials, and relevant websites, i.e., clinicaltrials.gov, clinicaltrialresults.com, tctmd.com, and esc365.escardio.org. We then searched conference proceedings from the American College of Cardiology, European Society of Cardiology, American Heart Association, and World Congress of Cardiology. We also performed a manual review of the reference lists of all included studies. References of recent narrative or systematic reviews, editorials, and meta-analyses were reviewed. We did not apply any restrictions on language, study period, or sample size. The last search was performed in November 2021.

### Study Selection

We included studies that met the following criteria: (1) include patients with AF and CKD (defined by CrCl <60 ml/min) treated by OACs (warfarin or DOACs, including rivaroxaban, dabigatran, apixaban, or edoxaban) for the prevention of stroke or thromboembolic events; (2) clearly provide more than one of the outcomes of interest separately in CKD patients; (3) present comparative results of outcomes among two or more OACs as an extractable form. We did not apply any exclusion criteria regarding the estimation equation of glomerular filtration rate (GFR). However, we primarily incorporated studies using the Cockcroft-Gault formula, and results from other formulae [e.g., Modification of Diet in Renal Disease (MDRD) or Chronic Kidney Disease Epidemiology Collaboration (CKD-EPI)] were only used if we could not extract any result from Cockcroft-Gault formula. We excluded studies conducted on AF patients on dialysis [defined as an end-stage renal disease (ESRD)]. We also excluded single-arm studies or non-randomized controlled studies (NRSs) that did not provide comparative results adjusted for confounding factors by multivariable-regression or propensity score (PS)-based methods (i.e., PS matching or inverse probability of treatment weighting). NRSs that did not include age, sex, major cardiovascular risk factors, or components of the CHA_2_DS_2_-VASc score in the multivariable regression model were also excluded. Unpublished subgroup data of CKD patients from the study by Lee et al. (24) were added (data provided in the Supplementary Materials). Two investigators, T-M Rhee and S-R Lee, independently screened the titles and abstracts from the search results, identified duplicated search results, reviewed full articles, and determined the eligibility of candidate studies. Disagreements between investigators were resolved by discussion with the other authors, E-K Choi and GYH Lip.

### Data Extraction and Quality Assessment

Summary data, as reported in the published articles, were used in the analysis. We used a standardized form to extract the comparative outcomes among OAC groups and detailed characteristics of each study. We assessed the quality of eligible studies using the Cochrane Risk-of-Bias tool for randomized trials (RoB 2) (28) for RCT and Risk Of Bias In Non-randomized Studies of Interventions (ROBINS-I) (29) for NRSs.

### Study Outcomes and Definitions

The outcomes of interest in the present study were (1) stroke or thromboembolism, (2) major bleeding, and (3) all-cause death at the longest available follow-up. Stroke or thromboembolism included both ischemic or hemorrhagic stroke and systemic arterial thromboembolism confirmed clinically or radiologically. The definition of major bleeding varied slightly from study to study but was mostly consistent with the International Society on Thrombosis and Haemostasis (ISTH) major bleeding criteria.

### Data Synthesis and Analysis

All results are presented according to the severity of renal impairment, i.e., all CKD with CrCl <60 mL/min, more than moderate CKD with CrCl <50 mL/min, and advanced CKD with CrCl <30 mL/min.

For pairwise direct comparisons for outcomes of interest between DOACs and warfarin, we established random-effects models and calculated pooled hazard ratios (HRs) with 95% confidence intervals (CIs) as summary statistics (30). Heterogeneity among studies was quantified using I^2^ statistics (30). Publication bias was assessed qualitatively using funnel plot asymmetry and quantitatively using Egger's and Begg's tests (30). To discriminate the significance of heterogeneity caused by including studies with different study types (RCT or NRS), various doses of DOAC (standard, reduced, or unspecified), and different GFR estimation equations (Cockcroft-Gault, MDRD, CKD-EPI, or unspecified), subgroup analyses were performed by (1) type of adjustment; (2) dose of DOAC; and (3) GFR estimation equation. The pooled HR and 95% CI in each subgroup was calculated and the heterogeneity was evaluated using I^2^ statistics.

For the network meta-analysis to compare outcomes across all the different OACs, we established a random-effects model based on a frequentist approach for multiple treatment comparisons (31). Pooled HRs and 95% CIs were presented as summary statistics and forest plots. A network league table summary was used to present all possible combinations of comparisons (32). The ranking of OACs from most to least beneficial for two outcomes, i.e., stroke or thromboembolism and major bleeding, was obtained by calculating P-scores from the frequentist treatment ranking method and simultaneously presented in the clustered ranking plot (33). Heterogeneity and inconsistency were evaluated by Q statistics, a network heat plot, and the network node-splitting method. Potential publication bias was assessed using a comparison-adjusted funnel plot and Egger's test (31, 32).

Two-sided *p* < 0.05 were considered statistically significant. We followed the Preferred Reporting Items for Systematic Reviews and Meta-Analyses (PRISMA) guideline (Supplementary Table 1) (34). The review protocol has been registered on the PROSPERO (CRD42021241718). Data were analyzed using Stata version 14.0 (StataCorp LP, College Station, Texas) and R version 4.0.4 (R Foundation for Statistical Computing, Vienna, Austria).

## Results

### Search Results and Study Selection

**Figure 1:**
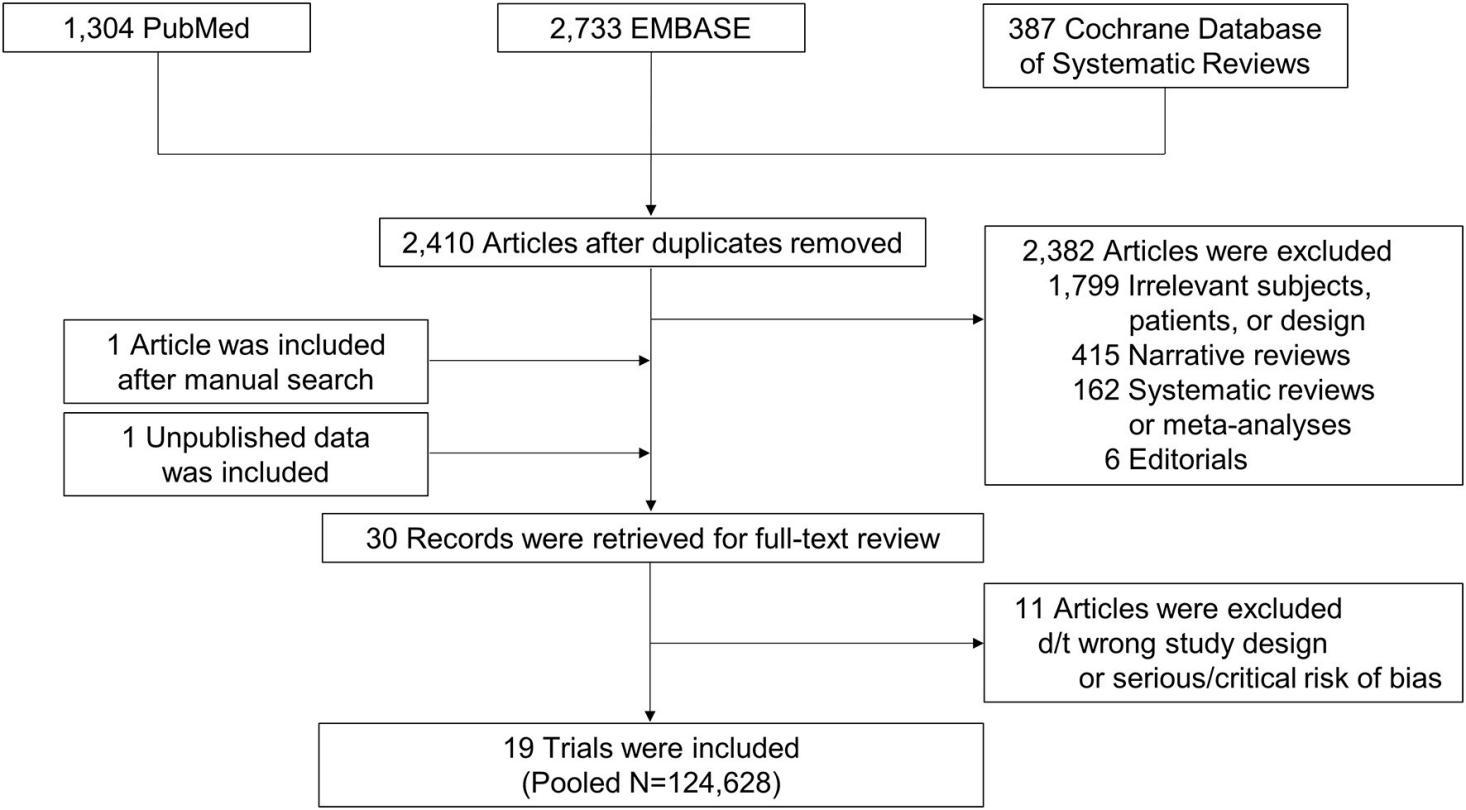
Study flow. Flow of search and study selection are presented.

We collected 2,410 articles and retrieved 30 studies for full-article review (Figure 1). Of these, 19 studies were included in the final analysis (6–10, 14–27). Five were subgroup analyses of previous RCTs (6–10). Direct comparisons between OACs were mostly conducted with warfarin as a reference group, while one study provided a direct comparison among DOACs (24). One study (14) did not report stroke or thromboembolism and 10 did not provide all-cause death (7, 14, 16, 17, 21–26).

### Characteristics of Included Trials

The period of study publications ranged from 2011 to 2020. Of the 14 NRSs, 10 used PS-based methods (PS matching or inverse probability of treatment weighting) (14–16, 21–27) and four used a multivariable regression model (Table 1 and Supplementary Table 2) (17–20). We incorporated 124,628 patients with AF and concomitant CKD (DOAC, *n* = 71,390; Warfarin, *n* = 53,238). The follow-up duration varied from 139 days to 5.5 years. The renal function of all pooled patients was CKD stage 3 or worse with CrCl <60 mL/min; five studies (6, 16, 17, 19, 25) provided outcomes for advanced CKD patients with CrCl <30 mL/min.

**Table 1:** Characteristics of studies selected for analysis.

**Study**	**Year**	**Study design**	**Adjustment method**	**DOAC group (*n*)**	**Warfarin group (*n*)**	**Renal function of enrolled patients**	**Duration of follow-up**	**Mean age (Y)**	**Male (%)**	**Mean CHA_**2**_DS_**2**_-VASc score**	**Mean HAS-BLED score**
ROCKET-AF (7)	2011	Randomized trial (CKD subgroup)	N/A	1,474	1,476	CrCl 30–50 mL/min	Median 590 days	73	60.3	3.48/3.46 (CHADS_2_)	NR
J-ROCKET AF (8)	2012	Randomized trial (CKD subgroup)	N/A	141	143	CrCl 30–50 mL/min	Up to 30 months	71.1	80.6	3.25 (CHADS_2_)	NR
ARISTOTLE (6)	2012	Randomized trial (CKD subgroup)	N/A	1,493	1,512	CrCl 25–50 mL/min	Median 1.8 years	77.6	46.7	4.4	2.2
RE-LY (9)	2014	Randomized trial (CKD subgroup)	N/A	2,428	1,126	CrCl 30–50 mL/min	Median 2 years	75.2	53.4	81.2% (CHADS_2_ ≥2)	NR
Hernandez et al. (14)	2015	Observational	Propensity-score based	428	2,536	CKD stage ≥3^*^	Median 177/228 days	75.1/75.6	42.1/41.0	80.9%/81.1% (CHADS_2_ ≥2)	NR
Lee et al. (20)	2015	Observational	Multivariate model-based	59	174	CKD stage ≥3	Median 596 days	71.9/69.3	37.7/34.1	≥2	NR
Engage Af-Timi 48 (10)	2016	Randomized trial (CKD subgroup)	N/A	1,379	1,361	CrCl 30–50 mL/min	Median 2.8 years	79	46	5.0	2.8
Shin et al. (21)	2018	Observational	Propensity-score based	1,122	1,122	CKD stage ≥3	Mean 1.2 years	73/72	53/54	4/4	2/2
Yu et al. (15)	2018	Observational	Propensity-score based	741	839	CrCl 30–50 mL/min	Median 5 months	68.2/68.3 (E60) 72.8/72.6 (E30)	63.3/63.0 (E60) 52.0/53.3 (E30)	4.2/4.2 (E60) 4.9/4.8 (E30)	NR
Coleman et al. (16)	2019	Observational	Propensity-score based	1,896	4,848	CKD stage ≥4^†^	Median 1.4 years	72/72	58.4/61.6	4	NR
Chan et al. (22)	2019	Observational	Propensity-score based	21,081	6,264	CKD stage ≥3	Up to 16 months	74.7	57	3.6	2.6
Bonnemeier et al. (23)	2019	Observational	Propensity-score based	4,164	7,002	CKD stage ≥3	Mean 381/221 days	76.9/77.2	45.5/50.8	4.6/4.5	3.5/3.4
Lee et al. (24)	2019	Observational	Propensity-score based	11,633	4,056	CKD stage ≥3	Up to 18 months	72/73	55/54	3.5/3.6	2.7/2.7
Chang et al. (17)	2019	Observational	Multivariate model-based	280	520	CKD stage ≥4	Mean 3.2 years	79.8/76.6	43.9/44.6	4.7/4.6	3.7/4.0
Laugesen et al. (18)	2019	Observational	Multivariate model-based	552	1,008	CKD stage ≥3	Up to 1 year	80.0/78.0	56.9/64.0	NR	NR
Makani et al. (19)	2020	Observational	Multivariate model-based	4,748	5,895	CKD stage ≥3	Median 3.4 years	75.7	50.0	≥2	NR
Weir et al. (25)	2020	Observational	Propensity-score based	781	1,536	CKD stage ≥4	Mean 389/370 days	79.9	39.5	4.5	3.5
Chan et al. (26)	2020	Observational	Propensity-score based	4,780	1,291	CKD stage ≥3	Up to 5.5 years	74.6/74.5	53.7/53.5	4.5/4.4	3.1/3.0
Wetmore et al. (27)	2020	Observational	Propensity-score based	12,210	10,529	CKD stage ≥3	Median 139 days	78/78	49/49	5.3/5.3	3.3/3.3

* *CKD stage ≥3 denotes estimated glomerular filtration rate below 60 mL/min/1.73 m^2^ as defined by the Kidney Disease: Improving Global Outcomes (KDIGO) classification*.

† *CKD stage ≥4 denotes estimated glomerular filtration rate below 30 mL/min/1.73 m^2^ as defined by the KDIGO classification*.

*CKD, chronic kidney disease; CrCl, creatinine clearance; DOAC, direct oral anticoagulant; E30, edoxaban 30 mg; E60, edoxaban 60 mg; N/A, not applicable; NR, not reported*.

### Assessment of Risk of Bias

The overall risk of bias was low for RCTs, except for one trial (8), which did not report a detailed randomization process. Although all NRSs had a moderate risk of bias due to their retrospective and observational nature, they showed low risk for most domains of bias (Supplementary Figure 1).

### Pairwise Comparison of DOAC vs. Warfarin in AF Patients With CKD

**Figure 2:**
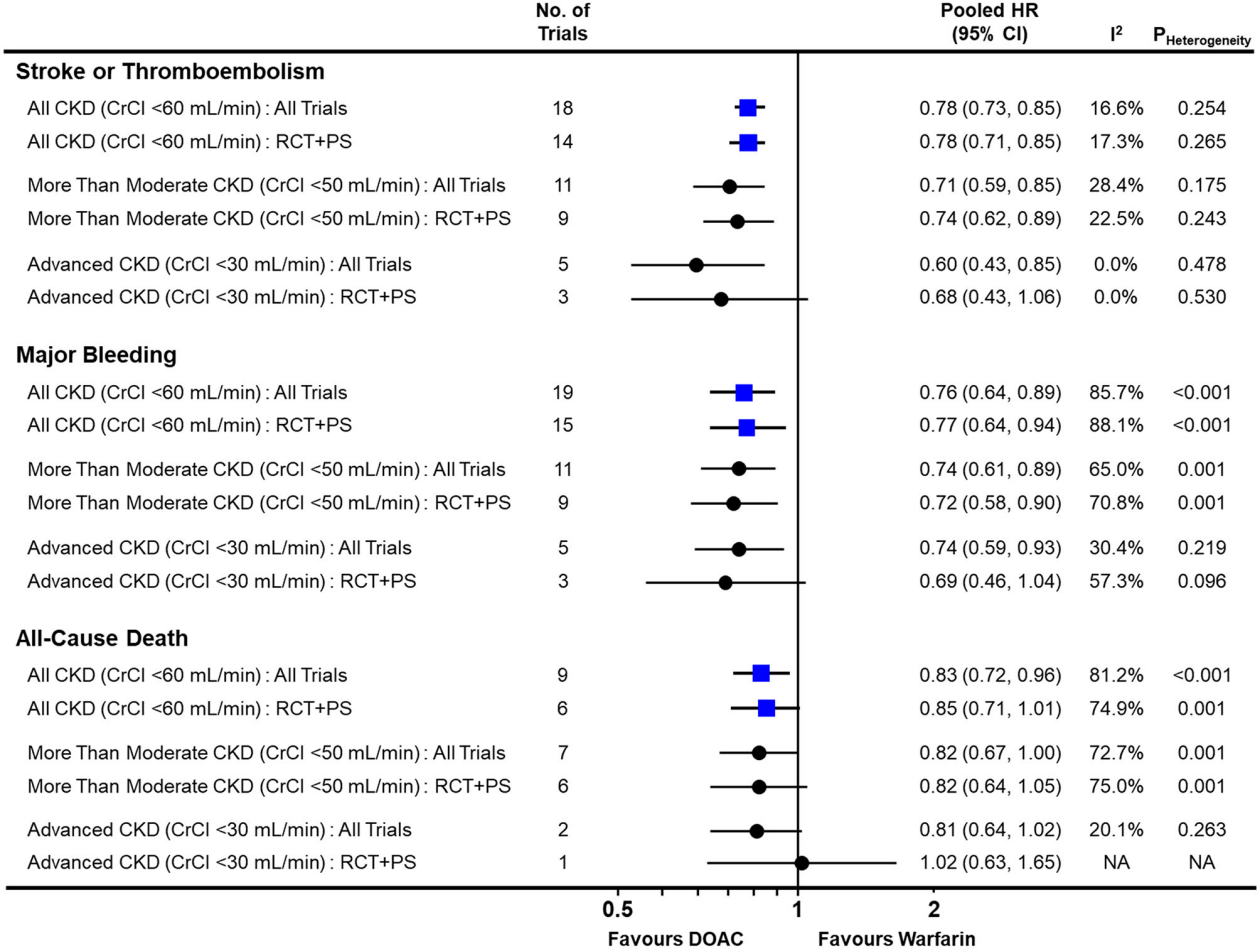
Comparison of pooled treatment effects of oral anticoagulants on clinical outcomes in atrial fibrillation patients with concomitant chronic kidney disease. Pooled HR and 95% CI, I^2^ and *P*-value for heterogeneity are presented for stroke or thromboembolism, major bleeding, and all-cause death according to the severity of renal impairment. CI, confidence interval; CKD, chronic kidney disease; CrCl, creatinine clearance; DOAC, direct oral anticoagulant; HR, hazard ratio; NA, not applicable; PS, propensity-score; RCT, randomized controlled trial.

In the pairwise meta-analysis with random-effects model (Figure 2 and Supplementary Figures 2–4), DOACs showed a significantly lower risk of stroke or thromboembolism [pooled HR = 0.78 (95% CI = 0.73–0.85), Heterogeneity I^2^ = 16.6%], major bleeding [pooled HR = 0.76 (95% CI = 0.64–0.89), I^2^ = 85.7%], and all-cause death [pooled HR = 0.83 (95% CI = 0.72–0.96), I^2^ = 81.2%] in the total CKD population when compared with warfarin. This was consistent, except for all-cause death, when pooling only RCTs and NRSs that used PS-based adjustment. Regardless of the severity of renal impairment, DOACs were significantly favored over warfarin for both stroke or thromboembolism and major bleeding.

In advanced CKD with CrCl <30 mL/min, DOACs significantly lowered the risk of stroke or thromboembolism [pooled HR = 0.60 (95% CI = 0.43–0.85), I^2^ = 0.0%] and major bleeding [pooled HR = 0.74 (95% CI = 0.59–0.93), I^2^ = 30.4%] when compared with warfarin. Additionally, they showed a tendency to lower the risk of all-cause death [pooled HR = 0.81 (95% CI = 0.64–1.02), I^2^ = 20.1%]. There was no evidence of publication bias for any of the outcomes (Supplementary Figure 5).

### Subgroup Analysis for Pairwise Meta-Analysis

**Figure 3:**
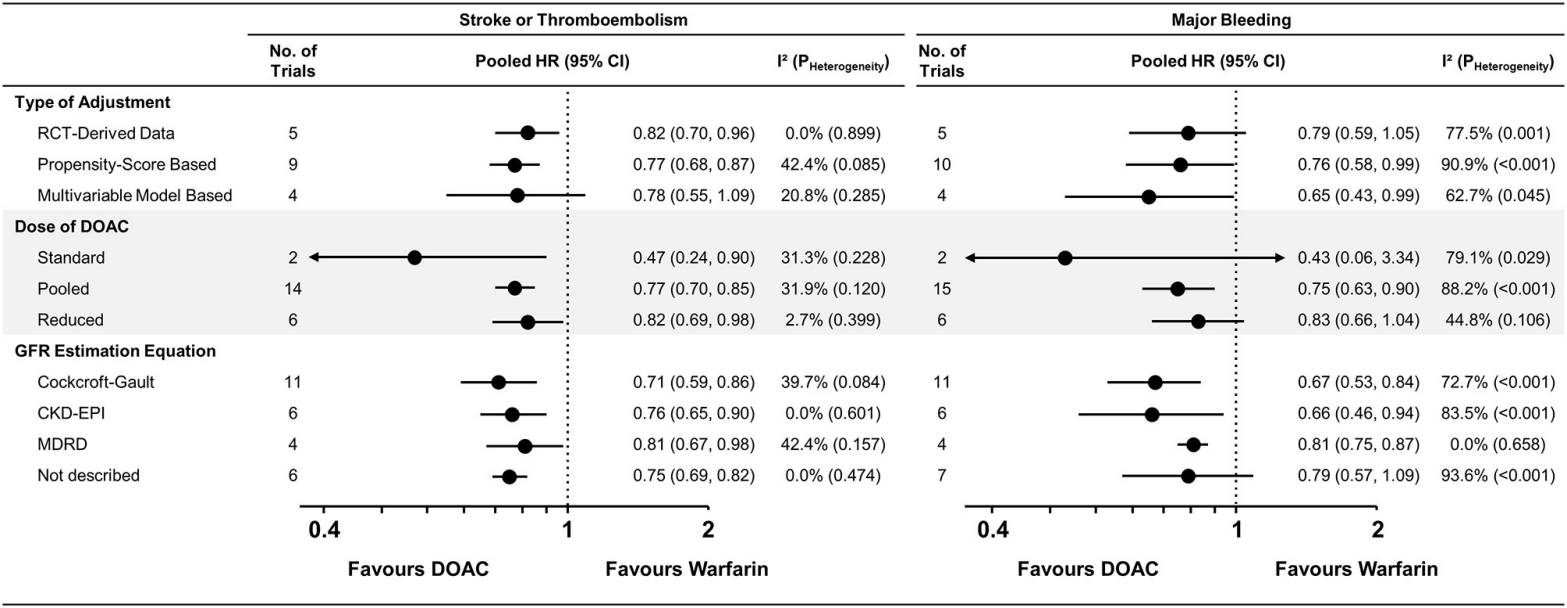
Subgroup analysis for stroke or thromboembolism and major bleeding. Subgroup analysis across various subgroups are presented. CKD-EPI, Chronic Kidney Disease Epidemiology Collaboration; GFR, glomerular filtration rate; MDRD, Modification of Diet in Renal Disease; CI, confidence interval; CKD, chronic kidney disease; CrCl, creatinine clearance; DOAC, direct oral anticoagulant; HR, hazard ratio; NA, not applicable; PS, propensity-score; RCT, randomized controlled trial.

The pairwise meta-analysis according to various subgroups was generally consistent with the main results (Figure 3 and Supplementary Figures 6–8). A similar trend was maintained in the RCTs, NRSs with PS-based adjustment, and NRSs with multivariable-model-based adjustment, while moderate heterogeneity in stroke or thromboembolism risk was observed among nine studies (15, 16, 21–27) that performed PS-based adjustment (I^2^ = 42.4%). A significant risk reduction for stroke or thromboembolism was still observed with a reduced dose of DOACs [pooled HR = 0.82 (95% CI = 0.69–0.98), I^2^ = 2.7%] when compared with warfarin. In the subgroups according to the GFR estimation equation, moderate heterogeneity was observed in studies using the Cockcroft-Gault (I^2^ = 39.7%) and MDRD equations (I^2^ = 42.4%), contrast to the studies using the CKD-EPI equation (I^2^ = 0.0%).

### Frequentist Network Meta-Analysis Comparing Efficacy and Safety of OACs for AF in Patients With CKD

**Figure 4:**
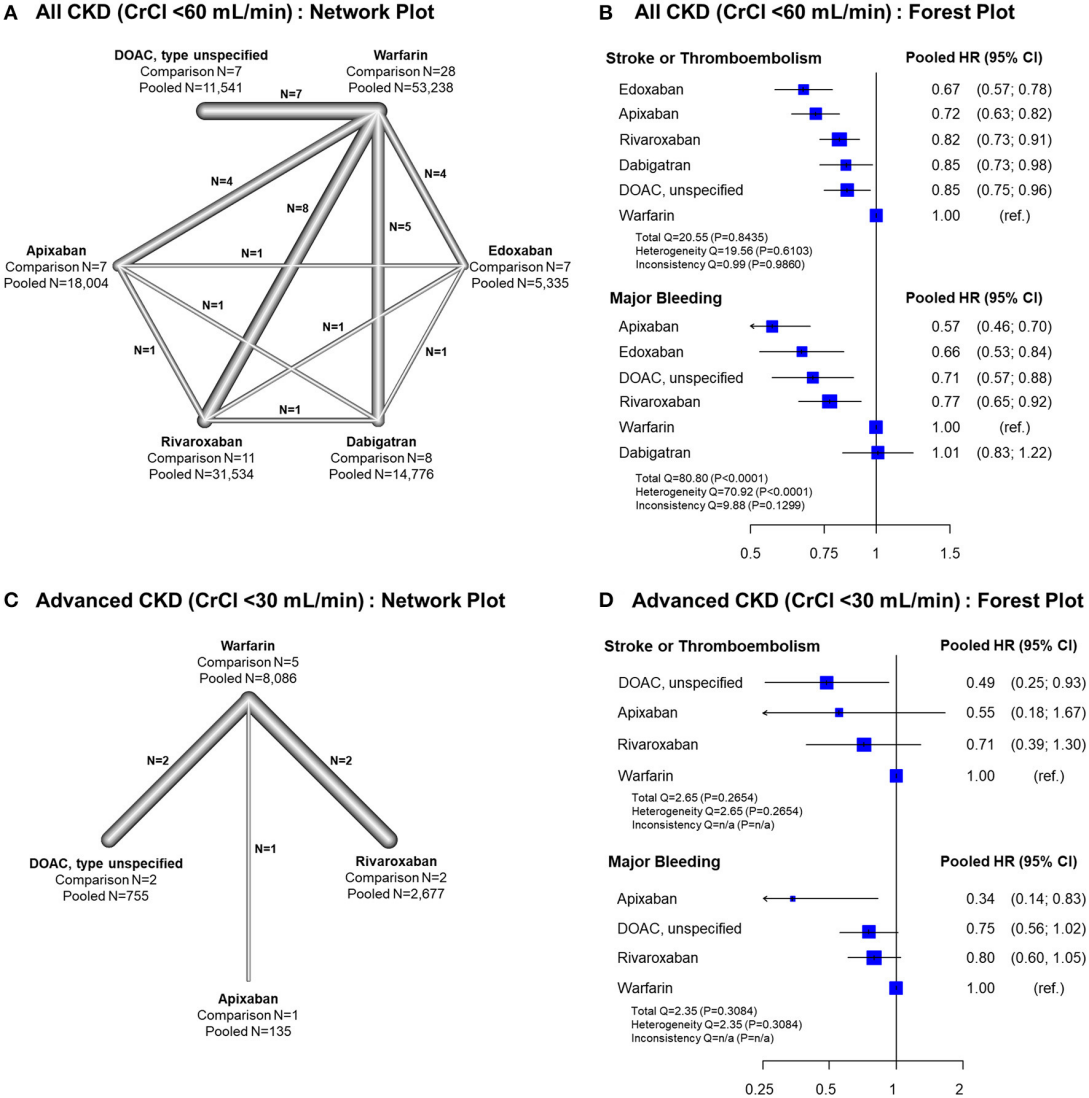
Results of network meta-analysis comparing safety and efficacy of oral anticoagulants in all CKD and advanced CKD patients. Results of frequentist network meta-analysis for all CKD patients with CrCl <60 mL/min, **(A)** network plot **(B)** forest plot, and for advanced CKD patients with CrCl <30 mL/min, **(C)** network plot, and **(D)** forest plot, are presented. ref., reference; CI, confidence interval; CKD, chronic kidney disease; CrCl, creatinine clearance; DOAC, direct oral anticoagulant; HR, hazard ratio; NA, not applicable; PS, propensity-score; RCT, randomized controlled trial.

In all CKD patients, all four DOACs showed significant risk reduction for stroke or thromboembolism with warfarin as a reference group (Figures 4A,B). Except dabigatran, all DOACs were significantly favored over warfarin in terms of major bleeding. Edoxaban showed a significantly lower risk of stroke or thromboembolism when compared with the other DOACs. For major bleeding, apixaban showed a significant benefit when compared with rivaroxaban and dabigatran, while dabigatran showed a significant increase of major bleeding risk when compared with all other DOACs (Table 2). A significant heterogeneity was observed for major bleeding (Heterogeneity Q = 70.92, *P* < 0.001), while there were possibilities of publication bias for both outcomes (Supplementary Figures 9, 10). In the advanced CKD group, (Figures 4C,D and Table 3) the risk of major bleeding was significantly lower in apixaban [pooled HR = 0.34 (95% CI = 0.14–0.83)] compared to warfarin.

**Table 2:** League table summary of network meta-analysis for oral anticoagulants in patients with chronic kidney disease (CrCl <60 mL/min).

	**Apixaban**	**Dabigatran**	**Edoxaban**	**DOAC, unspecified**	**Rivaroxaban**	**Warfarin**
Apixaban	-	0.56 (0.43, 0.73)	0.85 (0.64, 1.13)	0.80 (0.59, 1.09)	0.73 (0.57, 0.93)	0.57 (0.46, 0.70)
Dabigatran	0.85 (0.71, 1.01)	-	1.52 (1.15, 2.00)	1.43 (1.07, 1.92)	1.30 (1.03, 1.65)	1.01 (0.83, 1.23)
Edoxaban	1.07 (0.89, 1.29)	1.26 (1.04, 1.54)	-	0.94 (0.68, 1.29)	0.86 (0.66, 1.12)	0.66 (0.53, 0.84)
DOAC, unspecified	0.84 (0.70, 1.01)	0.99 (0.82, 1.20)	0.79 (0.64, 0.96)	-	0.91 (0.69, 1.21)	0.71 (0.57, 0.88)
Rivaroxaban	0.88 (0.76, 1.02)	1.04 (0.88, 1.22)	0.82 (0.69, 0.98)	1.04 (0.89, 1.23)	-	0.77 (0.65, 0.92)
Warfarin	0.72 (0.63, 0.82)	0.85 (0.73, 0.98)	0.67 (0.57, 0.78)	0.85 (0.75, 0.97)	0.82 (0.73, 0.91)	-
	Pooled HR and 95% CI for stroke or thromboembolism (first column as the reference group)				
	Pooled HR and 95% CI for major bleeding (first row as the reference group)				

*CI, confidence interval; CrCl, creatinine clearance; HR, hazard ratio; DOAC, direct oral anticoagulant*.

**Table 3:** League table summary of network meta-analysis for oral anticoagulants in patients with advanced chronic kidney disease (CrCl <30 mL/min).

	**Apixaban**	**Dabigatran**	**Edoxaban**	**DOAC, unspecified**	**Rivaroxaban**	**Warfarin**
Apixaban	-	Not available	Not available	0.45 (0.18, 1.15)	0.43 (0.17, 1.08)	0.34 (0.14, 0.83)
Dabigatran	Not available	-	Not available	Not available	Not available	Not available
Edoxaban	Not available	Not available	-	Not available	Not available	Not available
DOAC, unspecified	1.13 (0.31, 4.09)	Not available	Not available	-	0.95 (0.63, 1.43)	0.75 (0.56, 1.02)
Rivaroxaban	0.77 (0.22, 2.72)	Not available	Not available	0.68 (0.28, 1.64)	-	0.80 (0.60, 1.05)
Warfarin	0.55 (0.18, 1.67)	Not available	Not available	0.49 (0.25, 0.93)	0.71 (0.39, 1.30)	-
	Pooled HR and 95% CI for stroke or thromboembolism (first column as the reference group)				
	Pooled HR and 95% CI for major bleeding (first row as the reference group)				

*CI, confidence interval; CrCl, creatinine clearance; HR, hazard ratio; DOAC, direct oral anticoagulant*.

**Figure 5:**
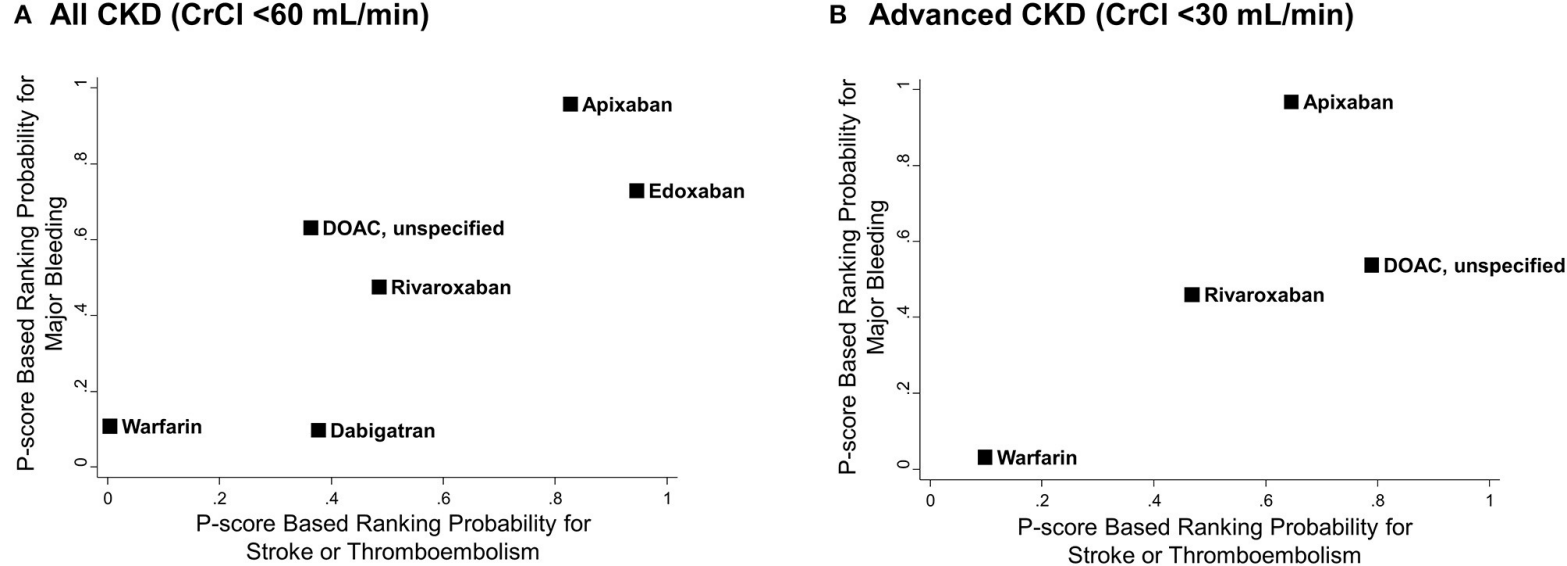
Clustered ranking plots of oral anticoagulants for stroke or thromboembolism and major bleeding. P-score based rankings of various oral anticoagulants for stroke or thromboembolism and major bleeding are plotted. **(A)** All CKD with CrCl <60 mL/min and **(B)** advanced CKD with CrCl <30 mL/min. CI, confidence interval; CKD, chronic kidney disease; CrCl, creatinine clearance; DOAC, direct oral anticoagulant; HR, hazard ratio; NA, not applicable; PS, propensity-score; RCT, randomized controlled trial.

Figure 5 illustrates the ranking probability of OACs for both outcomes by a clustered ranking plot. For the total CKD population, apixaban and edoxaban showed higher rank probabilities than other OACs for both stroke or thromboembolism (P-score for ranking probability, apixaban = 82.7% and edoxaban = 94.5%) and major bleeding (P-score, apixaban = 95.8% and edoxaban = 73.0%). Warfarin showed the lowest ranking probability (P-score for stroke or thromboembolism = 0.4% and for major bleeding = 10.8%). In the advanced CKD group, apixaban showed the highest rank for major bleeding (P-score = 96.9%), while it was the second-best strategy in terms of stroke prevention (P-score = 64.5%).

## Discussion

By incorporating RCTs as well as high-quality NRS data, we performed a comprehensive meta-analysis on the anticoagulation in AF patients with renal impairment. Our major findings can be summarized as follows. First, DOACs were better OAC treatment options for AF patients with concomitant CKD when compared with warfarin. They showed significantly lower risks of stroke or thromboembolism and major bleeding, regardless of the severity of renal impairment. Second, apixaban and edoxaban presented higher ranks than the other DOACs in terms of stroke or thromboembolism and major bleeding in the total CKD population. Apixaban remained the best treatment option in advanced CKD patients, particularly to reduce the risk of major bleeding.

### Optimal Anticoagulation Strategies in AF Patients With CKD

Although anticoagulant therapy in non-valvular AF lowers the risk of fatal stroke, bleeding, and mortality even for CKD patients (5, 35), starting OAC might be challenging in AF patients with CKD who are known to be associated with high risks of both thromboembolism and bleeding (3, 35–37). After the introduction of DOACs, a meta-analysis including pivotal RCTs showed consistent or accentuated clinical benefits of DOACs when compared with warfarin in this population (4).

In the present meta-analysis, we confirmed the superior safety and efficacy of DOACs compared with warfarin in all CKD patients with CrCl <60 mL/min, which was in line with previous evidence. Reduced mortality was also expected in the DOAC group. Our results may suggest that physicians should not compare DOACs vs. warfarin anymore; rather, they should consider which DOAC to use in AF patients with CKD.

### Efficacy and Safety of DOACs in Advanced CKD Patients With CrCl <30 mL/min

Data on the relative efficacy and safety of OACs in patients with advanced non-end stage CKD (stage 4 or worse without renal replacement therapy with CrCl <30 mL/min) are highly limited, mainly because these patients were excluded from pivotal RCTs except for the ARISTOTLE trial which covered 269 patients with CrCl 25–30 mL/min (6). By incorporating data from the recent observational studies covering the advanced CKD population (16, 17, 19, 25, 27), we found that DOACs significantly reduced the risk of stroke or thromboembolism as well as that of major bleeding compared to warfarin even in this population.

The increase of the area under the curve (AUC) for the plasma concentration of DOACs is predictable to some extent, except for dabigatran (13). In contrast, warfarin has a significantly suboptimal time in the therapeutic range as renal function worsens (36). Along with the possibility of extensive drug–drug interactions of warfarin, this may explain the superiority of DOACs shown in patients with advanced CKD. Our results may be an important cornerstone that can emphasize the necessity of a large-scale randomized trial comparing the efficacy and safety of each DOAC in advanced CKD patients. Furthermore, investigation to determine the optimal dosing strategy of each DOAC in this population is warranted.

### Comparison Among Different DOACs for AF Patients With CKD

Our network meta-analysis results showed that all four DOACs consistently showed significant risk reductions for stroke or thromboembolism and major bleeding compared with warfarin in AF patients with CKD, except dabigatran in terms of major bleeding. Among the DOACs, apixaban and edoxaban were ranked as the highest treatment recommendation. When compared with rivaroxaban and dabigatran, edoxaban showed significantly better efficacy in preventing stroke or thromboembolism, and apixaban significantly lowered the risk of major bleeding. Notably, dabigatran showed a risk of major bleeding similar to that of warfarin and thus showed significantly inferior results when compared with other DOACs. For patients with advanced CKD with CrCl <30 mL/min, we found that apixaban was the best DOAC treatment, especially in terms of reducing major bleeding risk. Although there is still a lack of evidence, these results are consistent with the consensus documented in current practical guidelines (13).

Differences in efficacy and safety according to DOAC types may be due to differences in the pharmacokinetic profiles of each DOAC. Apixaban has the lowest proportion of renal excretion; therefore, the AUC increase of plasma concentration is the most modest according to the decrease in renal function (13). The pharmacokinetic report from the ARISTOTLE substudy shows that the AUC of apixaban in the CrCl 25–30 mL/min patient group was similar to that of the group with CrCl 30–50 mL/min (6). The safety concern regarding high risk of major bleeding for dabigatran in CKD patients could also be explained by the excretion mostly dependent on the kidney. We found the best efficacy of edoxaban for the prevention of thromboembolism in the AF with CKD population. Further investigation is necessary for this novel finding. Better compliance of patients due to the once-daily regimen of edoxaban may have influenced the better outcomes, particularly in the observational study. The well-established dose reduction criteria of edoxaban may also explain the results of this study because the meticulous care of patients may have been possible in the edoxaban group.

### Study Limitations

This study has several limitations. First, it was a study-level meta-analysis; therefore, it was impossible to consider individual patient-level confounders. In addition, bias due to unmeasured or inaccessible confounding factors from observational studies could not be completely excluded. Second, although inconsistency between direct and indirect evidence was not found, evidence through direct comparison between DOACs was relatively scant, and currently, there is no head-to-head randomized trial for DOACs. Third, we tried to minimize heterogeneity following the incorporation of NRSs, but a significant level of heterogeneity was still observed in terms of major bleeding and all-cause death, requiring attention in interpretation for these outcomes. Nevertheless, the meta-analysis of the advanced CKD group showed negligible heterogeneity for both stroke or thromboembolism and major bleeding; thus, we assume that the heterogeneity issue minimally affected the core results of the present study. Fourth, the efficacy and safety of DOACs in the patient group requiring dialysis due to ESRD were not covered in this study. Fifth, there were studies in which the dose of DOAC was not reported and in which the proportion of different types of DOACs used was not described. Considering the variable effects of off-label dosing (38, 39), this may have increased the possible heterogeneity of the overall study results. Finally, we could not properly address the comparative efficacy and safety of off-label dosing of DOACs in AF with CKD patients in this study, which needs to be elucidated in future studies.

In conclusion, in patients with AF and CKD, DOACs were safer and more effective than warfarin regardless of severity of renal impairment. Among DOACs, apixaban and edoxaban presented higher rank probabilities compared to other DOACs as well as warfarin for both stroke prevention and a reduced risk of major bleeding. For advanced CKD patients with CrCl <30 mL/min, apixaban should be the first choice, especially in terms of safety.

## Data Availability Statement

The raw data supporting the conclusions of this article will be made available by the authors, without undue reservation.

## Author Contributions

All authors listed have made a substantial, direct, and intellectual contribution to the work and approved it for publication.

## Funding

This work was supported by the Korea Medical Device Development Fund grant funded by the Korea Government (the Ministry of Science and ICT, the Ministry of Trade, Industry and Energy, the Ministry of Health and Welfare, Republic of Korea, the Ministry of Food and Drug Safety) (Project Number: 202013B14), and by the Korea National Research Foundation funded by the Ministry of Education, Science and Technology (grant 2020R1F1A106740).

## Conflict of Interest

E-KC: Research grants or speaking fees from Bayer, BMS/Pfizer, Biosense Webster, Chong Kun Dang, Daiichi-Sankyo, Dreamtech Co., Ltd., Medtronic, Samjinpharm, Sanofi-Aventis, Seers Technology, Skylabs, and Yuhan; GL: Consultant and speaker for BMS/Pfizer, Boehringer Ingelheim and Daiichi-Sankyo. The remaining authors declare that the research was conducted in the absence of any commercial or financial relationships that could be construed as a potential conflict of interest.

## Publisher's Note

All claims expressed in this article are solely those of the authors and do not necessarily represent those of their affiliated organizations, or those of the publisher, the editors and the reviewers. Any product that may be evaluated in this article, or claim that may be made by its manufacturer, is not guaranteed or endorsed by the publisher.

## Abbreviations

AF: atrial fibrillation
CKD: chronic kidney disease
CrCl: creatinine clearance
DOAC: direct oral anticoagulant
ISTH: International Society on Thrombosis and Haemostasis
NRS: non-randomized studies
OAC: oral anticoagulant
PS: propensity score
RCT: randomized controlled trial.

## References

[1] Watanabe H Watanabe T Sasaki S Nagai K Roden DM Aizawa Y. Close bidirectional relationship between chronic kidney disease and atrial fibrillation: the Niigata preventive medicine study. Am Heart J. (2009) 158:629–36. 10.1016/j.ahj.2009.06.031 19781424

[2] Ding WY Gupta D Wong CF Lip GYH. Pathophysiology of atrial fibrillation and chronic kidney disease. Cardiovasc Res. (2020) 117:1046–59. 10.1093/cvr/cvaa258 32871005

[3] Olesen JB Lip GY Kamper AL Hommel K Køber L Lane DA . Stroke and bleeding in atrial fibrillation with chronic kidney disease. N Engl J Med. (2012) 367:625–35. 10.1056/NEJMoa1105594 22894575

[4] Ruff CT Giugliano RP Braunwald E Hoffman EB Deenadayalu N Ezekowitz MD . Comparison of the efficacy and safety of new oral anticoagulants with warfarin in patients with atrial fibrillation: a meta-analysis of randomised trials. Lancet. (2014) 383:955–62. 10.1016/S0140-6736(13)62343-0 24315724

[5] Hindricks G Potpara T Dagres N Arbelo E Bax JJ Blomström-Lundqvist C . 2020 Esc guidelines for the diagnosis and management of atrial fibrillation developed in collaboration with the european association for cardio-thoracic surgery (Eacts). Eur Heart J. (2021) 42:373–498. 10.1093/eurheartj/ehaa612 32860505

[6] Stanifer JW Pokorney SD Chertow GM Hohnloser SH Wojdyla DM Garonzik S . Apixaban versus warfarin in patients with atrial fibrillation and advanced chronic kidney disease. Circulation. (2020) 141:1384–92. 10.1161/CIRCULATIONAHA.119.044059 32160801

[7] Patel MR Mahaffey KW Garg J Pan G Singer DE Hacke W . Rivaroxaban versus warfarin in nonvalvular atrial fibrillation. N Engl J Med. (2011) 365:883–91. 10.1056/NEJMoa1009638 21830957

[8] Hori M Matsumoto M Tanahashi N Momomura S Uchiyama S Goto S . Rivaroxaban vs. Warfarin in Japanese patients with atrial fibrillation – the J-ROCKET AF study. Circ J. (2012) 76:2104–11. 10.1253/circj.CJ-12-0454 22664783

[9] Hijazi Z Hohnloser SH Oldgren J Andersson U Connolly SJ Eikelboom JW . Efficacy and safety of dabigatran compared with warfarin in relation to baseline renal function in patients with atrial fibrillation: a RE-LY (randomized evaluation of long-term anticoagulation therapy) trial analysis. Circulation. (2014) 129:961–70. 10.1161/CIRCULATIONAHA.113.003628 24323795

[10] Bohula EA Giugliano RP Ruff CT Kuder JF Murphy SA Antman EM . Impact of renal function on outcomes with edoxaban in the ENGAGE AF-TIMI 48 trial. Circulation. (2016) 134:24–36. 10.1161/CIRCULATIONAHA.116.022361 27358434

[11] Del-Carpio Munoz F Gharacholou SM Munger TM Friedman PA Asirvatham SJ Packer DL . Meta-analysis of renal function on the safety and efficacy of novel oral anticoagulants for atrial fibrillation. Am J Cardiol. (2016) 117:69–75. 10.1016/j.amjcard.2015.09.046 26698882

[12] Andò G Capranzano P. Non-vitamin K antagonist oral anticoagulants in atrial fibrillation patients with chronic kidney disease: a systematic review and network meta-analysis. Int J Cardiol. (2017) 231:162–9. 10.1016/j.ijcard.2016.11.303 28007305

[13] Steffel J Verhamme P Potpara TS Albaladejo P Antz M Desteghe L . The 2018 European heart rhythm association practical guide on the use of non-vitamin K antagonist oral anticoagulants in patients with atrial fibrillation. Eur Heart J. (2018) 39:1330–93. 10.1093/eurheartj/ehy136 29562325

[14] Hernandez I Baik SH Piñera A Zhang Y. Risk of bleeding with dabigatran in atrial fibrillation. JAMA Intern Med. (2015) 175:18–24. 10.1001/jamainternmed.2014.5398 25365537 PMC6608584

[15] Yu HT Yang PS Kim TH Jang E Kim D Uhm JS . Impact of renal function on outcomes with edoxaban in real-world patients with atrial fibrillation. Stroke. (2018) 49:2421–9. 10.1161/STROKEAHA.118.021387 30355093

[16] Coleman CI Kreutz R Sood NA Bunz TJ Eriksson D Meinecke AK . Rivaroxaban versus warfarin in patients with nonvalvular atrial fibrillation and severe kidney disease or undergoing hemodialysis. Am J Med. (2019) 132:1078–83. 10.1016/j.amjmed.2019.04.013 31054829

[17] Chang SH Wu CV Yeh YH Kuo CF Chen YL Wen MS . Efficacy and safety of oral anticoagulants in patients with atrial fibrillation and stages 4 or 5 chronic kidney disease. Am J Med. (2019) 132:1335–43.e6. 10.1016/j.amjmed.2019.06.006 31278930

[18] Laugesen EK Staerk L Carlson N Kamper AL Olesen JB Torp-Pedersen C . Non-vitamin K antagonist oral anticoagulants Vs. vitamin-K Antagonists In Patients With Atrial Fibrillation And Chronic Kidney Disease: A Nationwide Cohort Study. Thromb J. (2019) 17:21. 10.1186/s12959-019-0211-y 31736658 PMC6849210

[19] Makani A Saba S Jain SK Bhonsale A Sharbaugh MS Thoma F . Safety and efficacy of direct oral anticoagulants versus warfarin in patients with chronic kidney disease and atrial fibrillation. Am J Cardiol. (2020) 125:210–4. 10.1016/j.amjcard.2019.10.033 31780073

[20] Lee KH Park HW Cho JG Yoon NS Kim SS Kim MR . Comparison of non-vitamin K antagonist oral anticoagulants and warfarin on clinical outcomes in atrial fibrillation patients with renal dysfunction. Europace. (2015) 17 (Suppl. 2):ii69–75. 10.1093/europace/euv198 26842118

[21] Shin JI Secora A Alexander GC Inker LA Coresh J Chang AR . Risks and benefits of direct oral anticoagulants across the spectrum of Gfr among incident and prevalent patients with atrial fibrillation. Clin J Am Soc Nephrol. (2018) 13:1144–52. 10.2215/CJN.13811217 30002224 PMC6086708

[22] Chan YH Lee HF See LC Tu HT Chao TF Yeh YH . Effectiveness and safety of four direct oral anticoagulants in Asian patients with nonvalvular atrial fibrillation. Chest. (2019) 156:529–43. 10.1016/j.chest.2019.04.108 31103697

[23] Bonnemeier H Huelsebeck M Kloss S. Comparative effectiveness of rivaroxaban versus a vitamin K antagonist in patients with renal impairment treated for non-valvular atrial fibrillation in Germany - a retrospective cohort study. Int J Cardiol Heart Vasc. (2019) 23:100367. 10.1016/j.ijcha.2019.100367 31111087 PMC6510975

[24] Lee SR Choi EK Kwon S Han KD Jung JH Cha MJ . Effectiveness and safety of contemporary oral anticoagulants among Asians with nonvalvular atrial fibrillation. Stroke. (2019) 50:2245–9. 10.1161/STROKEAHA.119.025536 31208303

[25] Weir MR Ashton V Moore KT Shrivastava S Peterson ED Ammann EM. Rivaroxaban versus warfarin in patients with nonvalvular atrial fibrillation and stage IV-V chronic kidney disease. Am Heart J. (2020) 223:3–11. 10.1016/j.ahj.2020.01.010 32112872

[26] Chan YH Lee HF Li PR Liu JR Chao TF Wu LS . Effectiveness, safety, and major adverse limb events in atrial fibrillation patients with concomitant diabetes mellitus treated with non-vitamin K antagonist oral anticoagulants. Cardiovasc Diabetol. (2020) 19:63. 10.1186/s12933-020-01043-2 32404168 PMC7222472

[27] Wetmore JB Roetker NS Yan H Reyes JL Herzog CA. Direct-acting oral anticoagulants versus warfarin in medicare patients with chronic kidney disease and atrial fibrillation. Stroke. (2020) 51:2364–73. 10.1161/STROKEAHA.120.028934 32640949

[28] Sterne JAC Savović J Page MJ Elbers RG Blencowe NS Boutron I . Rob 2: a revised tool for assessing risk of bias in randomised trials. BMJ. (2019) 366:l4898. 10.1136/bmj.l4898 31462531

[29] Sterne JA Hernán MA Reeves BC Savović J Berkman ND Viswanathan M . Robins-I: a tool for assessing risk of bias in non-randomised studies of interventions. BMJ. (2016) 355:i4919. 10.1136/bmj.i4919 27733354 PMC5062054

[30] Chaimani A Mavridis D Salanti G. A hands-on practical tutorial on performing meta-analysis with stata. Evid Based Ment Health. (2014) 17:111–6. 10.1136/eb-2014-101967 25288685 PMC13404023

[31] White IR. Network meta-analysis. Stata J. (2015) 15:951–85. 10.1177/1536867X1501500403

[32] Shim S Yoon B-H Shin I-S Bae J-M. Network meta-analysis: application and practice using stata. Epidemiol Health. (2017) 39:e2017047. 10.4178/epih.e2017047 29092392 PMC5733388

[33] Chaimani A Salanti G. Visualizing assumptions and results in network meta-analysis: the network graphs package. Stata J. (2015) 15:905–50. 10.1177/1536867X1501500402

[34] Moher D Shamseer L Clarke M Ghersi D Liberati A Petticrew M . Preferred reporting items for systematic review and meta-analysis protocols (Prisma-P) 2015 statement. Syst Rev. (2015) 4:1. 10.1186/2046-4053-4-1 25554246 PMC4320440

[35] Bonde AN Lip GY Kamper AL Hansen PR Lamberts M Hommel K . Net clinical benefit of antithrombotic therapy in patients with atrial fibrillation and chronic kidney disease: a nationwide observational cohort study. J Am Coll Cardiol. (2014) 64:2471–82. 10.1016/j.jacc.2014.09.051 25500231

[36] Yang F Hellyer JA Than C Ullal AJ Kaiser DW Heidenreich PA . Warfarin utilisation and anticoagulation control in patients with atrial fibrillation and chronic kidney disease. Heart. (2017) 103:818–26. 10.1136/heartjnl-2016-309266 27852694 PMC5515726

[37] Potpara TS Ferro C Lip GYH Dan GA Lenarczyk R Mallamaci F . Management of atrial fibrillation in patients with chronic kidney disease in clinical practice: a joint European heart rhythm association (Ehra) and European Renal association/European dialysis and transplantation association (Era/Edta) physician-based survey. Europace. (2020) 22:496–505. 10.1093/europace/euz358 31965154

[38] Yao X Shah ND Sangaralingham LR Gersh BJ Noseworthy PA. Non-vitamin K antagonist oral anticoagulant dosing in patients with atrial fibrillation and renal dysfunction. J Am Coll Cardiol. (2017) 69:2779–90. 10.1016/j.jacc.2017.03.600 28595692

[39] Lee SR Lee YS Park JS Cha MJ Kim TH Park J . Label adherence for non-vitamin k antagonist oral anticoagulants in a prospective cohort of Asian patients with atrial fibrillation. Yonsei Med J. (2019) 60:277–84. 10.3349/ymj.2019.60.3.277 30799590 PMC6391519

