# Supplementary material for: Efficacy and Safety of Oral Anticoagulants for Atrial Fibrillation Patients With Chronic Kidney Disease: A Systematic Review and Meta-Analysis
Source: Front Cardiovasc Med. 2022 Jun 10;9:885548. doi: 10.3389/fcvm.2022.885548 (PMC9226375; doi:10.3389/fcvm.2022.885548)
Supplement: Supplementary file 1 [file Data_Sheet_1.docx]

**Supplementary Materials**

**1. Supplementary Methods**

**Search Strategies for PubMed, EMBASE, and Cochrane Database of Systematic Reviews**

**List of Excluded Studies and Reason for Exclusion**

**2. Supplementary Tables**

**Supplementary Table 1. Checklist of items to include when reporting a systematic review or meta-analysis (PRISMA guidelines)**

**Supplementary Table 2. Detailed Characteristics of Included Studies**

**3. Supplementary Figures**

**Supplementary Figure 1. Risk of Bias Assessment for Randomised Controlled Trials and Non-Randomised Studies**

**Supplementary Figure 2. Pooled Results for Stroke or Thromboembolism According to the Degree of Renal Impairment**

**Supplementary Figure 3. Pooled Results for Major Bleeding According to the Degree of Renal Impairment**

**Supplementary Figure 4. Pooled Results for All-Cause Death According to the Degree of Renal Impairment**

**Supplementary Figure 5. Results of Analysis for Publication Bias**

**Supplementary Figure 6. Subgroup Analysis According to the Method of Adjustment for Baseline Differences**

**Supplementary Figure 7. Subgroup Analysis According to the Dose of Non-Vitamin K Oral Anticoagulants**

**Supplementary Figure 8. Subgroup Analysis According to the Glomerular Filtration Rate Estimation Equation**

**Supplementary Figure 9. Validity of Frequentist Network Meta-Analysis for Stroke or Thromboembolism**

**Supplementary Figure 10. Validity of Frequentist Network Meta-Analysis for Major Bleeding**

**1. Supplementary Methods**

**Search strategies for PubMed, EMBASE, and Cochrane Database of Systematic Reviews**

| PubMed | | | EMBASE | | | Cochrane Database | | |
| --- | --- | --- | --- | --- | --- | --- | --- | --- |
| **#1** | atrial fibrillation[Title/Abstract] | 74972 | **#1** | 'atrial fibrillation':ti,ab,kw | 133687 | **#1** | (atrial fibrillation):ti,ab,kw | 12911 |
| **#2** | AF[Title/Abstract] | 43563 | **#2** | 'AF':ti,ab,kw | 82331 | **#2** | (AF):ti,ab,kw | 7314 |
| **#3** | Afib[Title/Abstract] | 383 | **#3** | 'Afib':ti,ab,kw | 1441 | **#3** | (Afib):ti,ab,kw | 91 |
| **#4** | A-fib[Title/Abstract] | 173 | **#4** | 'A-fib':ti,ab,kw | 501 | **#4** | (A-fib):ti,ab,kw | 28 |
| **#5** | #1 OR #2 OR #3 OR #4 | 90874 | **#5** | #1 OR #2 OR #3 OR #4 | 160654 | **#5** | #1 OR #2 OR #3 OR #4 | 14598 |
| **#6** | chronic kidney disease[Title/Abstract] | 53346 | **#6** | 'chronic kidney disease':ti,ab,kw | 87394 | **#6** | (chronic kidney disease):ti,ab,kw | 11872 |
| **#7** | CKD[Title/Abstract] | 31974 | **#7** | 'CKD':ti,ab,kw | 60022 | **#7** | (CKD):ti,ab,kw | 5507 |
| **#8** | renal failure[Title/Abstract] | 91041 | **#8** | 'renal failure':ti,ab,kw | 135759 | **#8** | (renal failure):ti,ab,kw | 18399 |
| **#9** | renal dysfunction[Title/Abstract] | 18370 | **#9** | 'renal dysfunction':ti,ab,kw | 29370 | **#9** | (renal dysfunction):ti,ab,kw | 4506 |
| **#10** | renal impairment[Title/Abstract] | 11662 | **#10** | 'renal impairment':ti,ab,kw | 20020 | **#10** | (renal impairment):ti,ab,kw | 3209 |
| **#11** | renal function[Title/Abstract] | 83836 | **#11** | 'renal function':ti,ab,kw | 133585 | **#11** | (renal function):ti,ab,kw | 21007 |
| **#12** | creatinine clearance[Title/Abstract] | 18568 | **#12** | 'creatinine clearance':ti,ab,kw | 27109 | **#12** | (creatinine clearance):ti,ab,kw | 6169 |
| **#13** | CrCl[Title/Abstract] | 1616 | **#13** | 'CrCl':ti,ab,kw | 3814 | **#13** | (CrCl):ti,ab,kw | 495 |
| **#14** | glomerular filtration rate[Title/Abstract] | 45686 | **#14** | 'glomerular filtration rate':ti,ab,kw | 66415 | **#14** | (glomerular filtration rate):ti,ab,kw | 9541 |
| **#15** | GFR[Title/Abstract] | 19726 | **#15** | 'GFR':ti,ab,kw | 38563 | **#15** | (GFR):ti,ab,kw | 8788 |
| **#16** | eGFR[Title/Abstract] | 64574 | **#16** | 'eGFR':ti,ab,kw | 123509 | **#16** | (eGFR):ti,ab,kw | 9257 |
| **#17** | #6 OR #7 OR #8 OR #9 OR #10 OR #11 OR #12 OR #13 OR #14 OR #15 OR #16 | 305235 | **#17** | #6 OR #7 OR #8 OR #9 OR #10 OR #11 OR #12 OR #13 OR #14 OR #15 OR #16 | 463941 | **#17** | #6 OR #7 OR #8 OR #9 OR #10 OR #11 OR #12 OR #13 OR #14 OR #15 OR #16 | 52708 |
| **#18** | anticoagulation[Title/Abstract] | 44093 | **#18** | 'anticoagulation':ti,ab,kw | 76344 | **#18** | (anticoagulation):ti,ab,kw | 6165 |
| **#19** | oral anticoagulant[Title/Abstract] | 6411 | **#19** | 'oral anticoagulant':ti,ab,kw | 10365 | **#19** | (oral anticoagulant):ti,ab,kw | 2094 |
| **#20** | oral anticoagulants[Title/Abstract] | 10894 | **#20** | 'oral anticoagulants':ti,ab,kw | 18577 | **#20** | (oral anticoagulants):ti,ab,kw | 2198 |
| **#21** | OAC[Title/Abstract] | 6185 | **#21** | 'OAC':ti,ab,kw | 9417 | **#21** | (OAC):ti,ab,kw | 438 |
| **#22** | OACs[Title/Abstract] | 491 | **#22** | 'OACs':ti,ab,kw | 918 | **#22** | (OACs):ti,ab,kw | 62 |
| **#23** | vitamin K antagonist[Title/Abstract] | 2927 | **#23** | 'vitamin K antagonist':ti,ab,kw | 4840 | **#23** | (vitamin K antagonist):ti,ab,kw | 594 |
| **#24** | vitamin K antagonists[Title/Abstract] | 4325 | **#24** | 'vitamin K antagonists':ti,ab,kw | 7347 | **#24** | (vitamin K antagonists):ti,ab,kw | 653 |
| **#25** | VKA[Title/Abstract] | 2018 | **#25** | 'VKA':ti,ab,kw | 4446 | **#25** | (VKA):ti,ab,kw | 475 |
| **#26** | VKAs[Title/Abstract] | 1116 | **#26** | 'VKAs':ti,ab,kw | 1869 | **#26** | (VKAs):ti,ab,kw | 149 |
| **#27** | warfarin[Title/Abstract] | 25123 | **#27** | 'warfarin':ti,ab,kw | 41927 | **#27** | (warfarin):ti,ab,kw | 4953 |
| **#28** | coumarin[Title/Abstract] | 11699 | **#28** | 'coumarin':ti,ab,kw | 13988 | **#28** | (coumarin):ti,ab,kw | 222 |
| **#29** | coumadin[Title/Abstract] | 1089 | **#29** | 'coumadin':ti,ab,kw | 2202 | **#29** | (coumadin):ti,ab,kw | 179 |
| **#30** | novel oral anticoagulant[Title/Abstract] | 336 | **#30** | 'novel oral anticoagulant':ti,ab,kw | 637 | **#30** | (novel oral anticoagulant):ti,ab,kw | 159 |
| **#31** | novel oral anticoagulants[Title/Abstract] | 1063 | **#31** | 'novel oral anticoagulants':ti,ab,kw | 1979 | **#31** | (novel oral anticoagulants):ti,ab,kw | 196 |
| **#32** | non-vitamin K antagonist oral anticoagulant[Title/Abstract] | 176 | **#32** | 'non-vitamin K antagonist oral anticoagulant':ti,ab,kw | 260 | **#32** | (non-vitamin K antagonist oral anticoagulant):ti,ab,kw | 76 |
| **#33** | non-vitamin K antagonist oral anticoagulants[Title/Abstract] | 872 | **#33** | 'non-vitamin K antagonist oral anticoagulants':ti,ab,kw | 1312 | **#33** | (non-vitamin K antagonist oral anticoagulants):ti,ab,kw | 128 |
| **#34** | nonvitamin K antagonist oral anticoagulant[Title/Abstract] | 26 | **#34** | 'nonvitamin K antagonist oral anticoagulant':ti,ab,kw | 33 | **#34** | (nonvitamin K antagonist oral anticoagulant):ti,ab,kw | 79 |
| **#35** | nonvitamin K antagonist oral anticoagulants[Title/Abstract] | 100 | **#35** | 'nonvitamin K antagonist oral anticoagulants':ti,ab,kw | 125 | **#35** | (nonvitamin K antagonist oral anticoagulants):ti,ab,kw | 126 |
| **#36** | NOAC[Title/Abstract] | 1481 | **#36** | 'NOAC':ti,ab,kw | 3389 | **#36** | (NOAC):ti,ab,kw | 237 |
| **#37** | NOACs[Title/Abstract] | 1821 | **#37** | 'NOACs':ti,ab,kw | 3728 | **#37** | (NOACs):ti,ab,kw | 205 |
| **#38** | direct oral anticoagulant[Title/Abstract] | 1040 | **#38** | 'direct oral anticoagulant':ti,ab,kw | 1625 | **#38** | (direct oral anticoagulant):ti,ab,kw | 421 |
| **#39** | direct oral anticoagulants[Title/Abstract] | 3152 | **#39** | 'direct oral anticoagulants':ti,ab,kw | 5380 | **#39** | (direct oral anticoagulants):ti,ab,kw | 598 |
| **#40** | DOAC[Title/Abstract] | 1671 | **#40** | 'DOAC':ti,ab,kw | 3361 | **#40** | (DOAC):ti,ab,kw | 196 |
| **#41** | DOACs[Title/Abstract] | 1969 | **#41** | 'DOACs':ti,ab,kw | 3769 | **#41** | (DOACs):ti,ab,kw | 214 |
| **#42** | rivaroxaban[Title/Abstract] | 5625 | **#42** | 'rivaroxaban':ti,ab,kw | 11740 | **#42** | (rivaroxaban):ti,ab,kw | 1697 |
| **#43** | dabigatran[Title/Abstract] | 5085 | **#43** | 'dabigatran':ti,ab,kw | 10336 | **#43** | (dabigatran):ti,ab,kw | 1034 |
| **#44** | apixaban[Title/Abstract] | 3640 | **#44** | 'apixaban':ti,ab,kw | 7803 | **#44** | (apixaban):ti,ab,kw | 936 |
| **#45** | edoxaban[Title/Abstract] | 1544 | **#45** | 'edoxaban':ti,ab,kw | 2746 | **#45** | (edoxaban):ti,ab,kw | 564 |
| **#46** | #18 OR #19 OR #20 OR #21 OR #22 OR #23 OR #24 OR #25 OR #26 OR #27 OR #28 OR #29 OR #30 OR #31 OR #32 OR #33 OR #34 OR #35 OR #36 OR #37 OR #38 OR #39 OR #40 OR #41 OR #42 OR #43 OR #44 OR #45 | 88775 | **#46** | #18 OR #19 OR #20 OR #21 OR #22 OR #23 OR #24 OR #25 OR #26 OR #27 OR #28 OR #29 OR #30 OR #31 OR #32 OR #33 OR #34 OR #35 OR #36 OR #37 OR #38 OR #39 OR #40 OR #41 OR #42 OR #43 OR #44 OR #45 | 142927 | **#46** | #18 OR #19 OR #20 OR #21 OR #22 OR #23 OR #24 OR #25 OR #26 OR #27 OR #28 OR #29 OR #30 OR #31 OR #32 OR #33 OR #34 OR #35 OR #36 OR #37 OR #38 OR #39 OR #40 OR #41 OR #42 OR #43 OR #44 OR #45 | 12615 |
| **#47** | #5 AND #17 AND #46 | 1304 | **#47** | #5 AND #17 AND #46 | 2733 | **#47** | #5 AND #17 AND #46 | 390 |
|  |  |  |  |  |  | **#48** | trials | 387 |

**List of Excluded Studies and Reason for Exclusion**

| No. | Title | Author | Journal (Year) | Main Reason for Exclusion |
| --- | --- | --- | --- | --- |
| 1 | Novel Oral Anticoagulants and the Risk of Major Hemorrhage in Elderly Patients With Chronic Kidney Disease: A Nested Case-Control Study | Harel Z et al. | Can J Cardiol (2016) | The study only provided odds ratios for summary measures. |
| 2 | Renal function and risk of stroke and bleeding in patients undergoing catheter ablation for atrial fibrillation: Comparison between uninterrupted direct oral anticoagulants and warfarin administration | Yanagisawa S et al. | Heart Rhythm (2018) | The investigators assessed that the study had serious risk of bias in terms of bias due to selection of participants. |
| 3 | Relationship between the Renal Function and Adverse Clinical Events in Patients with Atrial Fibrillation: A Japanese Multicenter Registry Substudy | Yuzawa Y et al. | J Clin Med (2020) | The investigators assessed that the study had serious risk of bias in terms of bias due to confounding. |
| 4 | Oral anticoagulation and cardiovascular outcomes in patients with atrial fibrillation and chronic kidney disease in Asian Population, Data from the COOL-AF Thailand registry | Chantrarat T et al. | Int J Cardiol (2021) | The investigators assessed that the study had critical risk of bias in terms of bias due to confounding, bias due to selection of participants, and bias in selection of the reported result. |
| 5 | Safety and Efficacy of Apixaban Versus Warfarin in Patients With Advanced Chronic Kidney Disease | Schafer JH et al. | Ann Pharmacother (2018) | The study did not provide any adjusted result for the baseline differences and possible confounding factors. The investigators assessed that the study had critical risk of bias in terms of bias due to confounding. |
| 6 | Safety and effectiveness of rivaroxaban and warfarin in moderate-to-advanced CKD: real world data | Di Lullo L et al. | J Nephrol (2018) | The study did not provide any adjusted result for the baseline differences and possible confounding factors. The investigators assessed that the study had critical risk of bias in terms of bias due to confounding. |
| 7 | Real-world 2-year outcome of atrial fibrillation treatment with dabigatran, apixaban, and rivaroxaban in patients with and without chronic kidney disease | Godino C et al. | Intern Emerg Med (2019) | The study did not provide any adjusted result for the baseline differences and possible confounding factors. The investigators assessed that the study had critical risk of bias in terms of bias due to confounding. |
| 8 | Efficacy and safety of direct oral anticoagulants in patients with atrial fibrillation and chronic kidney disease | Bhatia HS et al. | Pacing Clin Electrophysiol (2019) | The study did not provide any adjusted result for the baseline differences and possible confounding factors. The investigators assessed that the study had critical risk of bias in terms of bias due to confounding. |
| 9 | Characterizing the Safety Profile of Apixaban Versus Warfarin in Moderate to Severe Chronic Kidney Disease at a Veterans Affairs Hospital | Herndon K et al. | Ann Pharmacother (2020) | The study did not provide any adjusted result for the baseline differences and possible confounding factors. The investigators assessed that the study had critical risk of bias in terms of bias due to confounding. |
| 10 | Safety and Efficacy of Direct Oral Anticoagulants for Atrial Fibrillation in Patients with Renal Impairment | Jang SM et al. | Pharmacy (2020) | The study did not provide any adjusted result for the baseline differences and possible confounding factors. The study only provided cross-sectional data. The investigators assessed that the study had critical risk of bias in terms of bias due to confounding. |
| 11 | Efficacy and Safety of Non-Vitamin K Antagonist Oral Anticoagulants in Patients With Atrial Fibrillation and Chronic Kidney Disease Stage G4: A Single-Center Experience | Heleniak Z et al. | J Cardiovasc Pharmacol (2020) | The study did not provide any adjusted result for the baseline differences and possible confounding factors. The investigators assessed that the study had critical risk of bias in terms of bias due to confounding. |

**2. Supplementary Tables**

**Supplementary Table 1. Checklist of items to include when reporting a systematic review or meta-analysis (PRISMA guidelines)**

| Section/topic | # | Checklist item | Reported on page # |
| --- | --- | --- | --- |
| **TITLE** | | | |
| Title | 1 | Identify the report as a systematic review, meta-analysis, or both. | #1-2 |
| **ABSTRACT** | | | |
| Structured summary | 2 | Provide a structured summary including, as applicable: background; objectives; data sources; study eligibility criteria, participants, and interventions; study appraisal and synthesis methods; results; limitations; conclusions and implications of key findings; systematic review registration number. | #3-5 |
| **INTRODUCTION** | | | |
| Rationale | 3 | Describe the rationale for the review in the context of what is already known. | #6 |
| Objectives | 4 | Provide an explicit statement of questions being addressed with reference to participants, interventions, comparisons, outcomes, and study design (PICOS). | #6 |
| **METHODS** | | | |
| Protocol and registration | 5 | Indicate if a review protocol exists, if and where it can be accessed (e.g., Web address), and, if available, provide registration information including registration number. | #7 |
| Eligibility criteria | 6 | Specify study characteristics (e.g., PICOS, length of follow-up) and report characteristics (e.g., years considered, language, publication status) used as criteria for eligibility, giving rationale. | #7 |
| Information sources | 7 | Describe all information sources (e.g., databases with dates of coverage, contact with study authors to identify additional studies) in the search and date last searched. | #7 |
| Search | 8 | Present full electronic search strategy for at least one database, including any limits used, such that it could be repeated. | #7 and Supplementary Material |
| Study selection | 9 | State the process for selecting studies (i.e., screening, eligibility, included in systematic review, and, if applicable, included in the meta-analysis). | #7-8 |
| Data collection process | 10 | Describe method of data extraction from reports (e.g., piloted forms, independently, in duplicate) and any processes for obtaining and confirming data from investigators. | #7-8 |
| Data items | 11 | List and define all variables for which data were sought (e.g., PICOS, funding sources) and any assumptions and simplifications made. | #7-8 |
| Risk of bias in individual studies | 12 | Describe methods used for assessing risk of bias of individual studies (including specification of whether this was done at the study or outcome level), and how this information is to be used in any data synthesis. | #8 |
| Summary measures | 13 | State the principal summary measures (e.g., risk ratio, difference in means). | #8 |
| Synthesis of results | 14 | Describe the methods of handling data and combining results of studies, if done, including measures of consistency (e.g., I^2^) for each meta-analysis. | #9 |
| Risk of bias across studies | 15 | Specify any assessment of risk of bias that may affect the cumulative evidence (e.g., publication bias, selective reporting within studies). | #9 |
| Additional analyses | 16 | Describe methods of additional analyses (e.g., sensitivity or subgroup analyses, meta-regression), if done, indicating which were pre-specified. | #9 |
| RESULTS | | | |
| Study selection | 17 | Give numbers of studies screened, assessed for eligibility, and included in the review, with reasons for exclusions at each stage, ideally with a flow diagram. | #10 and Figure 1 |
| Study characteristics | 18 | For each study, present characteristics for which data were extracted (e.g., study size, PICOS, follow-up period) and provide the citations. | #10, Table 1, and Supplementary Table 2 |
| Risk of bias within studies | 19 | Present data on risk of bias of each study and, if available, any outcome-level assessment (see Item 12). | #11 and Supplementary Figure 1 |
| Results of individual studies | 20 | For all outcomes considered (benefits or harms), present, for each study: (a) simple summary data for each intervention group and (b) effect estimates and confidence intervals, ideally with a forest plot. | #11, Figure 2 and Supplementary Figure 2 to 4 |
| Synthesis of results | 21 | Present results of each meta-analysis done, including confidence intervals and measures of consistency. | #11-12 and Figure 2 |
| Risk of bias across studies | 22 | Present results of any assessment of risk of bias across studies (see Item 15). | #11 and Supplementary Figure 5 |
| Additional analysis | 23 | Give results of additional analyses, if done (e.g., sensitivity or subgroup analyses, meta-regression [see Item 16]). | #11-12, Figure 3 to 5, and Supplementary Figure 6 to 10 |
| DISCUSSION | | | |
| Summary of evidence | 24 | Summarize the main findings including the strength of evidence for each main outcome; consider their relevance to key groups (e.g., health care providers, users, and policy makers). | #13-15 |
| Limitations | 25 | Discuss limitations at study and outcome level (e.g., risk of bias), and at review level (e.g., incomplete retrieval of identified research, reporting bias). | #15-16 |
| Conclusions | 26 | Provide a general interpretation of the results in the context of other evidence, and implications for future research. | #16 |
| FUNDING | | | |
| Funding | 27 | Describe sources of funding for the systematic review and other support (e.g., supply of data); role of funders for the systematic review. | #16-17 |

**Supplementary Table 2. Detailed Characteristics of Included Studies**

| **Study** | **Year** | **Inclusion criteria** | **Exclusion criteria** | **Primary outcomes** | **Secondary outcomes** | **Link to the public trial registry** |
| --- | --- | --- | --- | --- | --- | --- |
| **ROCKET-AF^7^** | 2011 | Documented atrial fibrillation on 2 separate occasions within 6 months before screening  History of a prior stroke, transient ischemic attack or non-neurologic systemic embolism believed to be cardiac in origin, or at least two of the following risk factors: heart failure, hypertension, age 75 years or greater, diabetes mellitus | Significant mitral stenosis  Transient atrial fibrillation caused by a reversible disorder  Active internal bleeding  Severe disabling stroke  History of intracranial bleeding  Hemorrhagic disorders | Efficacy : composite of stroke/non-CNS systemic embolism  Safety : major/non-major clinically relevant bleeding | Death, myocardial infarction | Clinicaltrials.gov, NCT00403767 |
| **J-ROCKET AF^8^** | 2012 | 20 years or older,  Japanese male or female,  Non- valvular atrial fibrillation documented by ECG,  Patients with a risk of stroke and non-CNS systemic embolism | Significant mitral stenosis,  Patients in whom anticoagulants are contraindicated | Safety : composite of major and non-major clinically relevant bleeding  Efficacy : composite of all-cause stroke and non-CNS systemic embolism | A composite of stroke, systemic embolism, and vascular death and a composite of stroke, systemic embolism, vascular death, and MI. Individual components of the composite secondary endpoints. | Clinicaltrials.gov, NCT00494871 |
| **ARISTOTLE^6^** | 2012 | Patients had to have AF or flutter at enrolment or at least two episodes of AF or flutter documented by electrocardiography at least 2 weeks apart in the 12 months before enrolment. In addition, at least one of the following risk factors for stroke was required: age ≥75 years; prior stroke, transient ischaemic attack, or systemic embolism; symptomatic heart failure within 3 months or left ventricular ejection fraction of no more than 40%; diabetes mellitus; hypertension requiring pharmacological treatment. | AF due to a reversible cause, moderate or severe mitral stenosis, conditions other than AF that required anticoagulation such as prosthetic heart valve, stroke within 7 days, need for aspirin >165 mg a day or both aspirin and clopidogrel, and severe renal insufficiency [serum creatinine >2.5 mg/dL (221 mmol/L) or calculated creatinine clearance <25 mL/min]. | Efficacy : Stroke or systemic embolism  Safety : ISTH major bleeding | All-cause death, myocardial infarction | Clinicaltrials.gov, NCT00412984 |
| **RE-LY^9^** | 2014 | Patients with AF who had ≥1 additional risk factor for stroke | Estimated GFR (eGFR) <30 mL/min according to Cockcroft-Gault | Efficacy : fatal and nonfatal stroke (ischemic, hemorrhagic, or unspecified) or systemic embolism.  Safety : major bleeding. | All-cause mortality | Clinicaltrials.gov, NCT00262600 |
| **Hernandez et al.^14^** | 2015 | Patients with newly diagnosed AF who filled prescriptions for dabigatran or warfarin within 2 months of the first diagnosis | Those who filled prescriptions for dabigatran and warfarin during the first 2 months after diagnosis | Major bleeding | Any bleeding |  |
| **Lee et al.^20^** | 2015 | AF patients who had a CHA_2_DS_2_-VASc score >2 and received anticoagulants according to current guidelines | No specific exclusion criteria | Composite of death, hospitalization, and new-onset strokes | Myocardial infarction, composite of major and minor bleeding by ISTH criteria |  |
| **ENGAGE AF-TIMI 48^10^** | 2016 | 21 years of age or older; male or female.  Able to provide written informed consent.  History of documented AF within the prior 12 months  A moderate to high risk of stroke, as defined by CHADS2 index score of at least 2 | Transient atrial fibrillation secondary to other reversible disorders  Subjects with moderate or severe mitral stenosis, unresected atrial myxoma, or a mechanical heart valve  Subjects with any contraindication for anticoagulant agents;  Subjects with conditions associated with high risk of bleeding or have known or suspected hereditary or acquired bleeding disorders  Females of childbearing potential including the following:  - Females with a history of tubal-ligation  - Females less than 2 years post-menopausal | Composite of stroke and systemic embolic events | Death, myocardial infarction, major/minor bleeding | Clinicaltrials.gov, NCT00781391 |
| **Shin et al.^21^** | 2018 | Diagnosis of atrial fibrillation who filled at least one prescription for anticoagulants and had a serum creatinine measured between Oct 19, 2010 and Feb 2, 2017. | No specific exclusion criteria | Bleeding, ischemic stroke | Described in the primary outcomes part |  |
| **Yu et al.^15^** | 2018 | Patients with prevalent AF who were aged >18 years | (1) those with valvular AF (with a diagnosis of mitral stenosis or prosthetic heart valves, and insurance claims for valve replacement or valvuloplasty; n=40 748), (2) those ever underwent catheter ablation (N=11 760), (3) those ever diagnosed as end-stage renal disease (N=14 988), (4) no OAC or OAC use <30 days (n=627 876), or (5) OAC use for acute coronary syndrome or deep vein thrombosis prophylaxis (N=4092). (6) Patients with other NOACs (dabigatran, rivaroxaban, and apixaban) (n=31 108). | Stroke or systemic embolism, major bleeding, death from any cause | Intracranial bleeding, gastrointestinal bleeding, myocardial infarction, admission for heart failure |  |
| **Coleman et al.^16^** | 2019 | adult patients with ≥12 months of continuous medical and prescription insurance coverage before index oral anticoagulant initiation, were oral anticoagulant-naı¨ve during the 12 months before the day of the first qualifying rivaroxaban or warfarin dispensing, ≥2 inpatient or outpatient ICD codes in any position for atrial fibrillation (ICD-10 = I48) | Patients with valvular heart disease, Patients with alternate indications for full-dose anticoagulation during the baseline period | Combination of stroke or systemic embolism | Major bleeding |  |
| **Chan et al.^22^** | 2019 | Patients diagnosed with AF and prescribed for DOACs or warfarin | Patients who took more than one DOAC during their treatment course  Patients diagnosed with end-stage renal disease, DVT, pulmonary embolism, joint replacement therapy, or valvular AF (eg, the diagnosis of mitral stenosis or valvular surgery) up to 6 months prior to the index date. | Ischemic stroke or systemic embolism  Major bleeding | Fatal ischemic stroke/systemic embolism, acute myocardial infarction |  |
| **Bonnemeier et al.^23^** | 2019 | New users of rivaroxaban or phenprocoumon in patients with nonvalvular AF and renal impairment, aged more than 18 years | Patients with evidence of cancer within the baseline period | Effectiveness : Ischemic stroke  Safety : intracranial hemorrhage | Combined endpoint of ischemic stroke and ICH |  |
| **Lee et al.^24^** | 2019 | OAC naïve nonvalvular AF patients treated with warfarin or 4 NOACs | 1) Patients <20 years of age 2) Patients diagnosed with valvular AF, pulmonary embolism or deep vein thrombosis 3) Patients received joint replacement 4) Patients diagnosed with ESRD | Ischemic stroke, intracranial hemorrhage, gastrointestinal bleeding, major bleeding | Described in the primary outcomes part |  |
| **Chang et al.^17^** | 2019 | 1) New diagnosis of atrial fibrillation  2) Patients had an eGFR measurement within 12 months before the initiation of anticoagulants or given index date and the eGFR <30 mL/min/1.73m2 (also known as stages 4 and 5 chronic kidney disease) | 1) those who had had a prescription of anticoagulant for less than 28 days; 2) those who had had a prolonged admission (>30 days) after the atrial fibrillation diagnosis; 3) those who had had a stroke or thromboembolism within 7 days of atrial fibrillation diagnosis; 4) those who had had received renal transplant before the atrial fibrillation diagnosis; 5) those who died before assigned index date; and 6) those who had switched oral anticoagulants during the study period. | 1) admissions with a primary diagnosis of major bleeding of gastrointestinal, intracranial, or urogenital tract  2) admissions with a primary diagnosis of ischemic stroke, transient ischemic attack, or systemic thromboembolism | Fatal outcome (death due to any cause) |  |
| **Laugesen et al.^18^** | 2019 | All Danish citizens with a prior diagnosis of AF and CKD who initiated OAC between 22nd Aug 2011 and 30th Jun 2017. | Valvular AF, defined as mechanical heart valve or rheumatic heart disease; Patients on dialysis; Patients initiated on edoxaban | Major bleeding  Stroke/thromboembolism | Myocardial infarction  All-cause mortality |  |
| **Makani et al.^19^** | 2020 | Nonvalvular AF, a CHA2DS2-VASc score of ≥2, and were prescribed either warfarin or DOAC. | Those who were not on anticoagulation | All-cause mortality  Bleeding requiring hospitalization  Stroke | Described in the primary outcomes part |  |
| **Weir et al.^25^** | 2020 | Adult patients with nonvalvular AF and stage IV-V CKD who initiated use of rivaroxaban or warfarin  (1) history of HER activity ≥183 days prior to index and ≥1 health care encounter recorded in the EHR during the 183 days prior to index; (2) diagnosed with AF and no evidence of valvular AF (ie, no diagnosed mitral stenosis or prior heart valve replacement); (3) diagnosed with CKD (stage IV, stage V, end-stage renal disease, or CKD unspecified); (4) confirmation of stage IV-V CKD based on CrCl b30 mL/ min estimated from serum creatinine using the Cockcroft-Gault equation14 and/or evidence of dialysis; (5) no history of kidney transplant; (6) no evidence of other potential common indications for anticoagulation (eg, venous thromboembolism, hip/knee replacement surgery); (7) no prior OAC use documented during the 183 days prior to index; (8) no study outcome events coded on the index date or 30 days prior; and (9) no death date recorded prior to index (imposed as a data quality measure). | No specific exclusion criteria | Hospitalization for ischemic stroke or systemic embolism | Hospitalization for major bleeding |  |
| **Chan et al.^26^** | 2020 | AF patients treated with oral anticoagulants after Jun 1, 2012. | Patients who took more than one NOAC type during their treatment course  Patients with diagnoses indicating deep vein thrombosis or pulmonary embolism, mitral stenosis, post valvular surgery, or joint replacement therapy within 6 months before the index date  Patients with end-stage renal disease | Effectiveness : ischemic stroke/systemic embolism, acute myocardial infarction, and major adverse cardiac events  Safety : Intracranial hemorrhage, major gastrointestinal bleeding, and all major bleeding events | Major lower limb outcomes : acute or chronic limb ischemia requiring revascularization procedures, lower limb amputation, and major adverse limb events |  |
| **Wetmore et al.^27^** | 2020 | Patients who used an anticoagulant between Jan 1, 2011 and Dec 31, 2017. | Patients who used anticoagulants within 1 year before the index date (ie, washout period).  Patients with other potential indications for anticoagulation, such as hip or knee replacement surgery or a venous thromboembolic event within the previous 60 days.  Patients with any history of specific valvular heart diseases or end-stage renal disease from Jan 1, 2010, on, and patients without CKD stage 3, 4, or 5 in the year preceding the index date. | Ischemic stroke and systemic embolism  Major bleeding | Described in the primary outcomes part |  |

**3. Supplementary Figures**

**Supplementary Figure 1. Risk of Bias Assessment for Randomised Controlled Trials and Non-Randomised Studies**

**
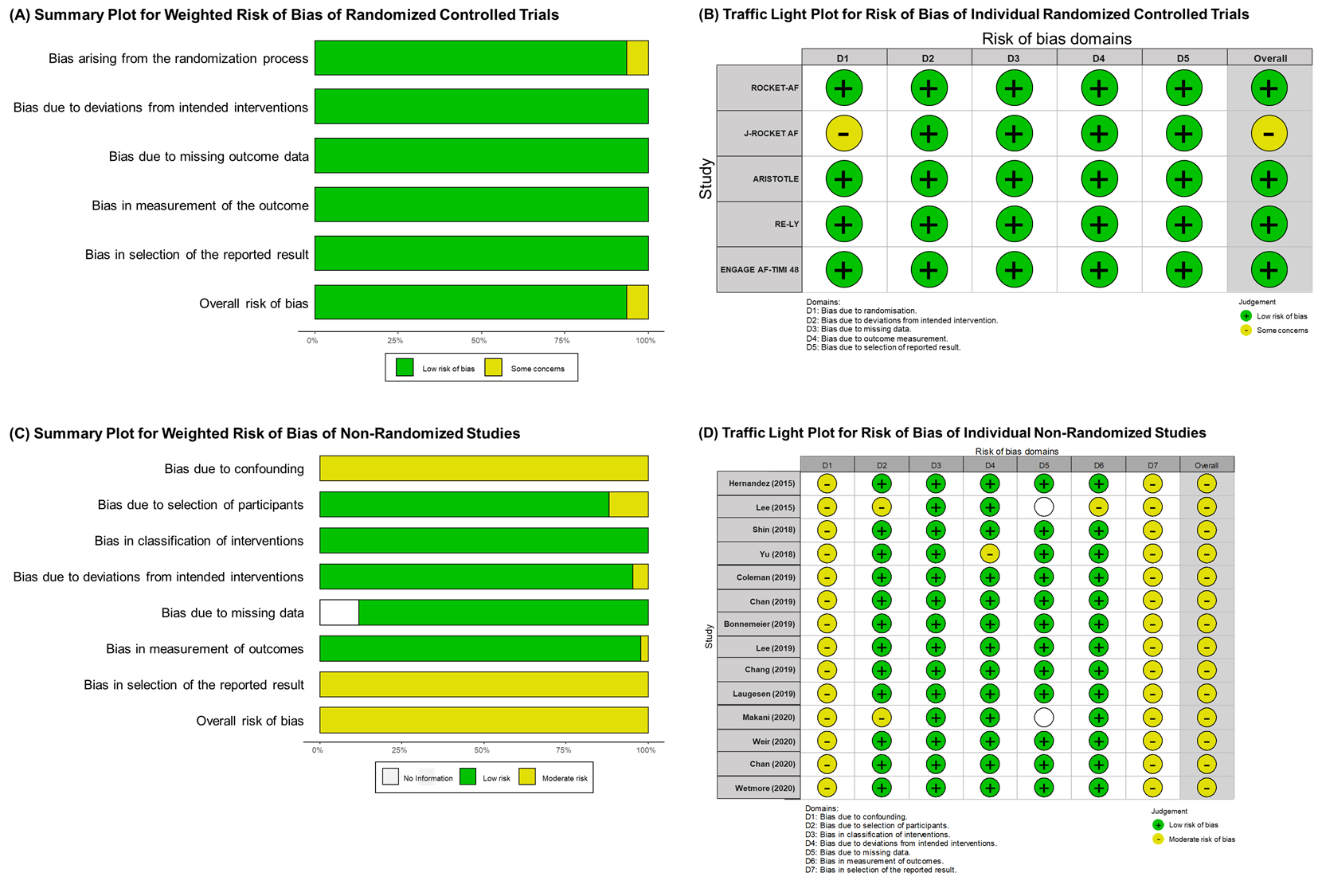
**

**Supplementary Figure 2. Pooled Results for Stroke or Thromboembolism According to the Degree of Renal Impairment**

**
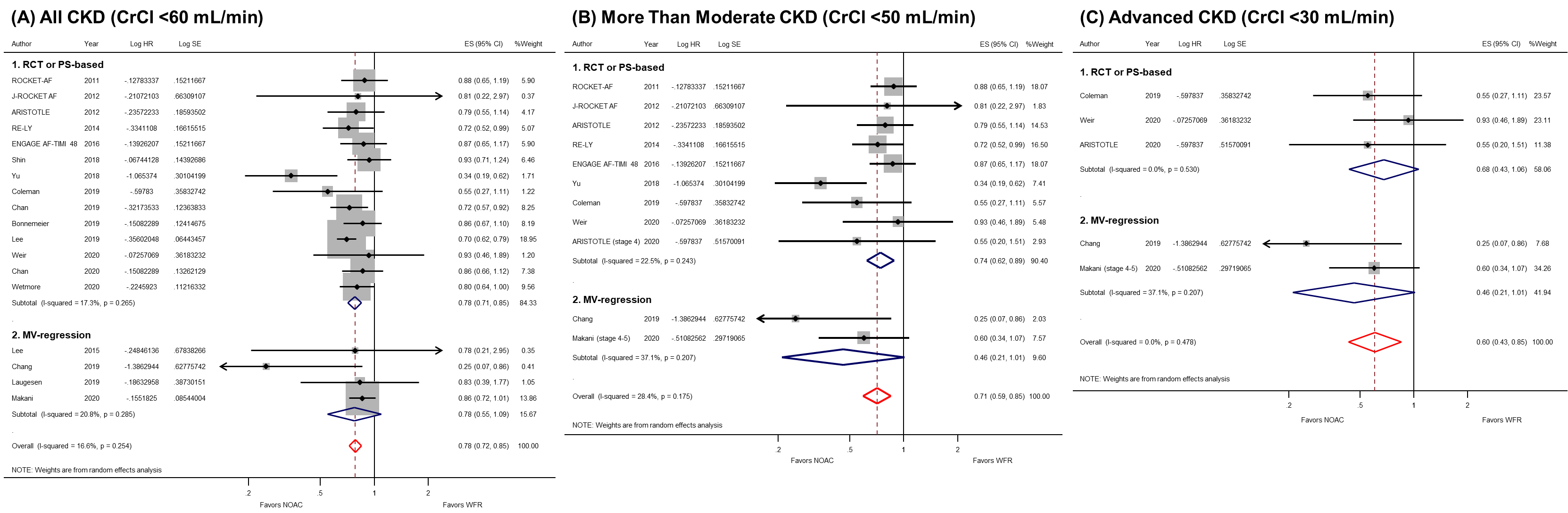
**

**Supplementary Figure 3. Pooled Results for Major Bleeding According to the Degree of Renal Impairment**

**
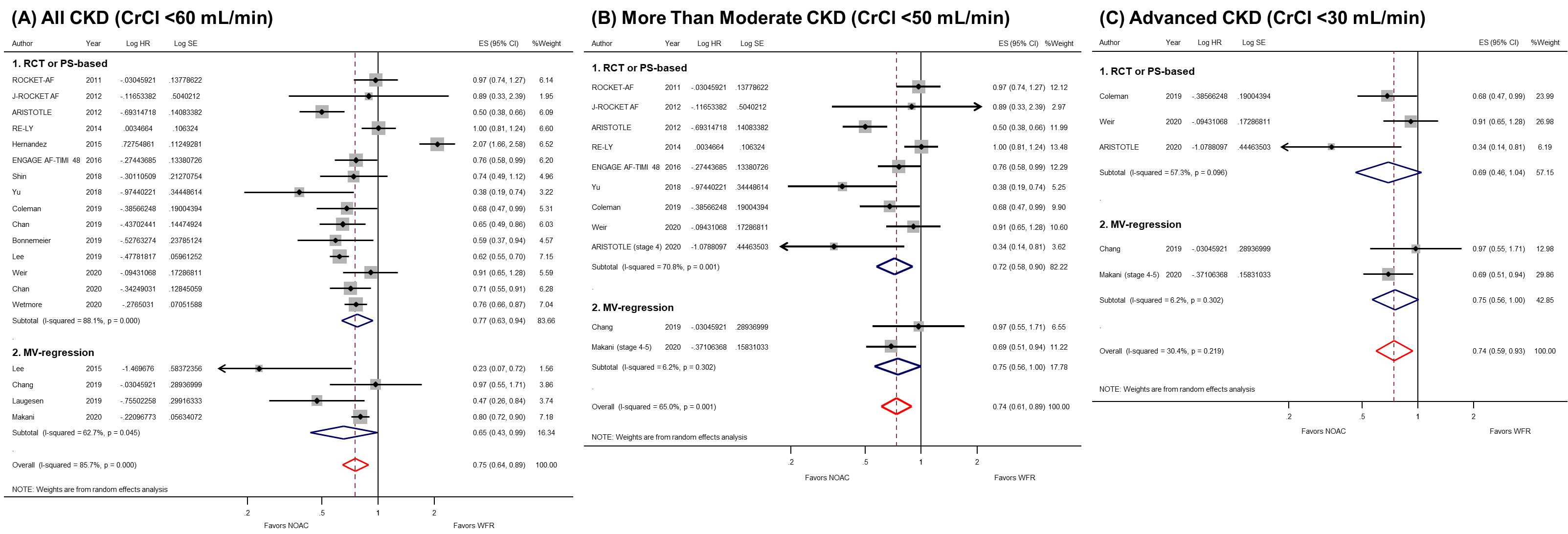
**

**Supplementary Figure 4. Pooled Results for All-Cause Death According to the Degree of Renal Impairment**

**
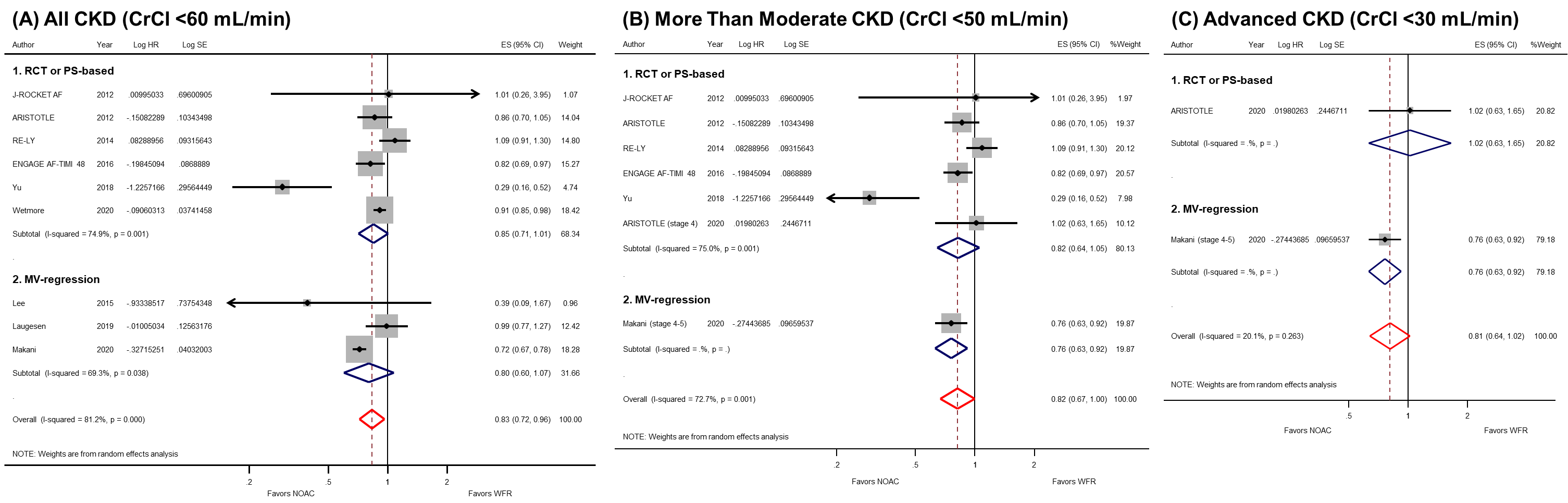
**

**Supplementary Figure 5. Results of Analysis for Publication Bias**

**
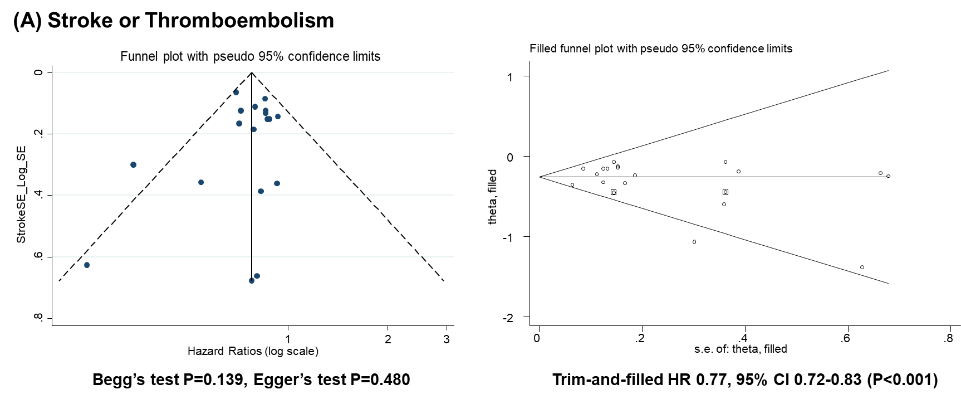
**

**
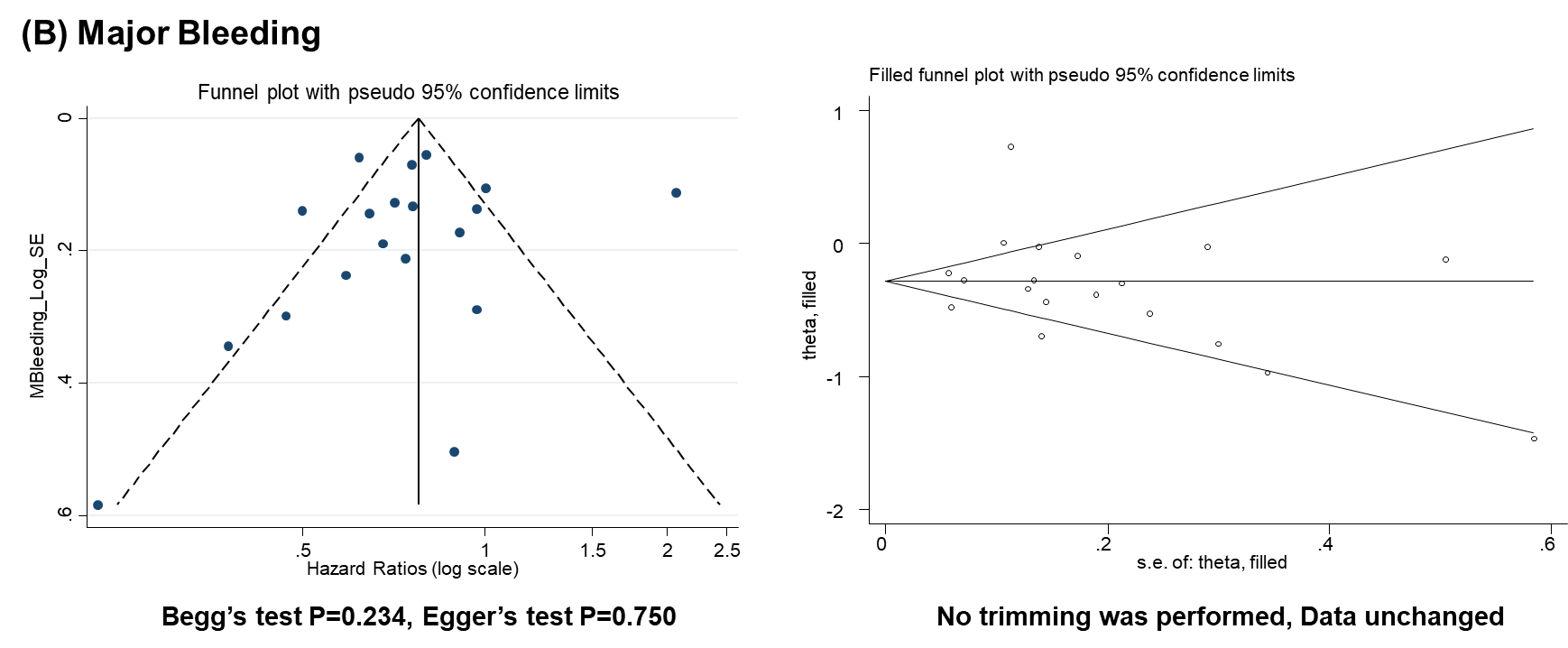
**

**
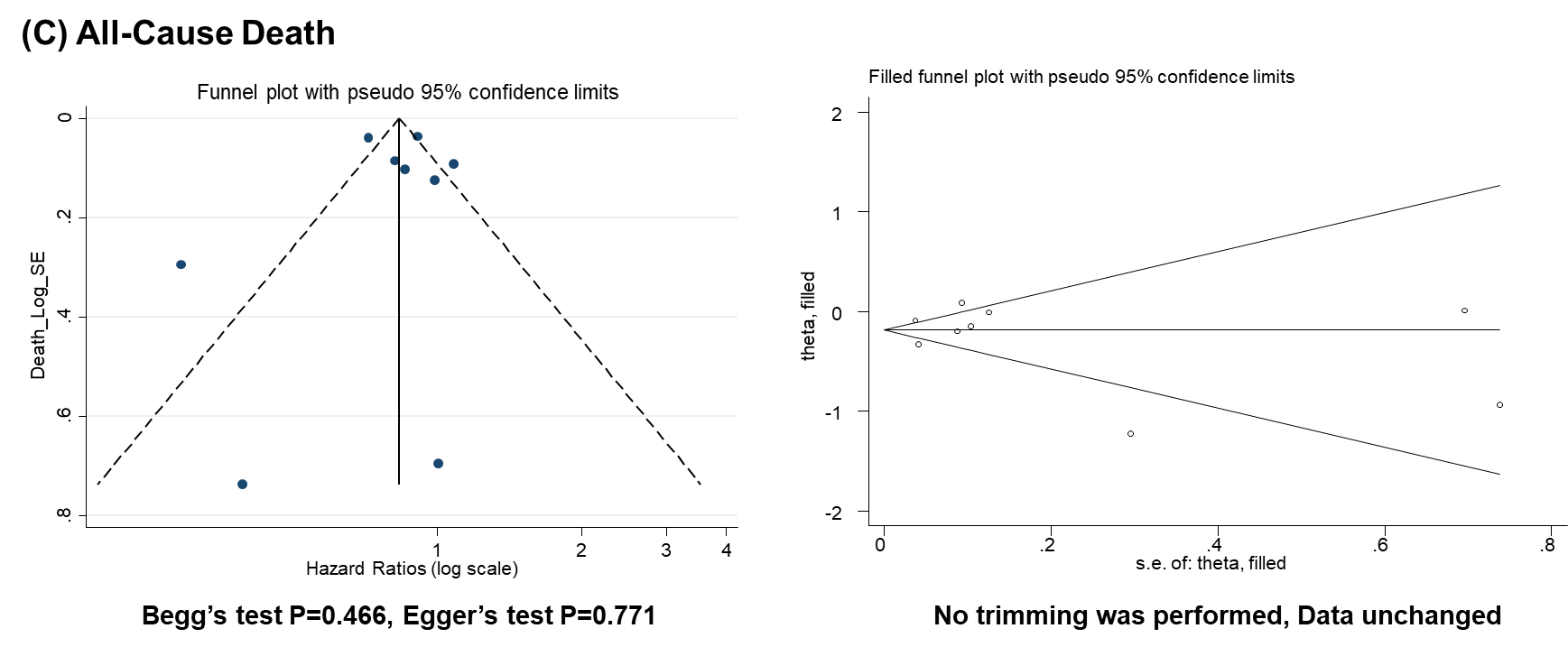
**

**
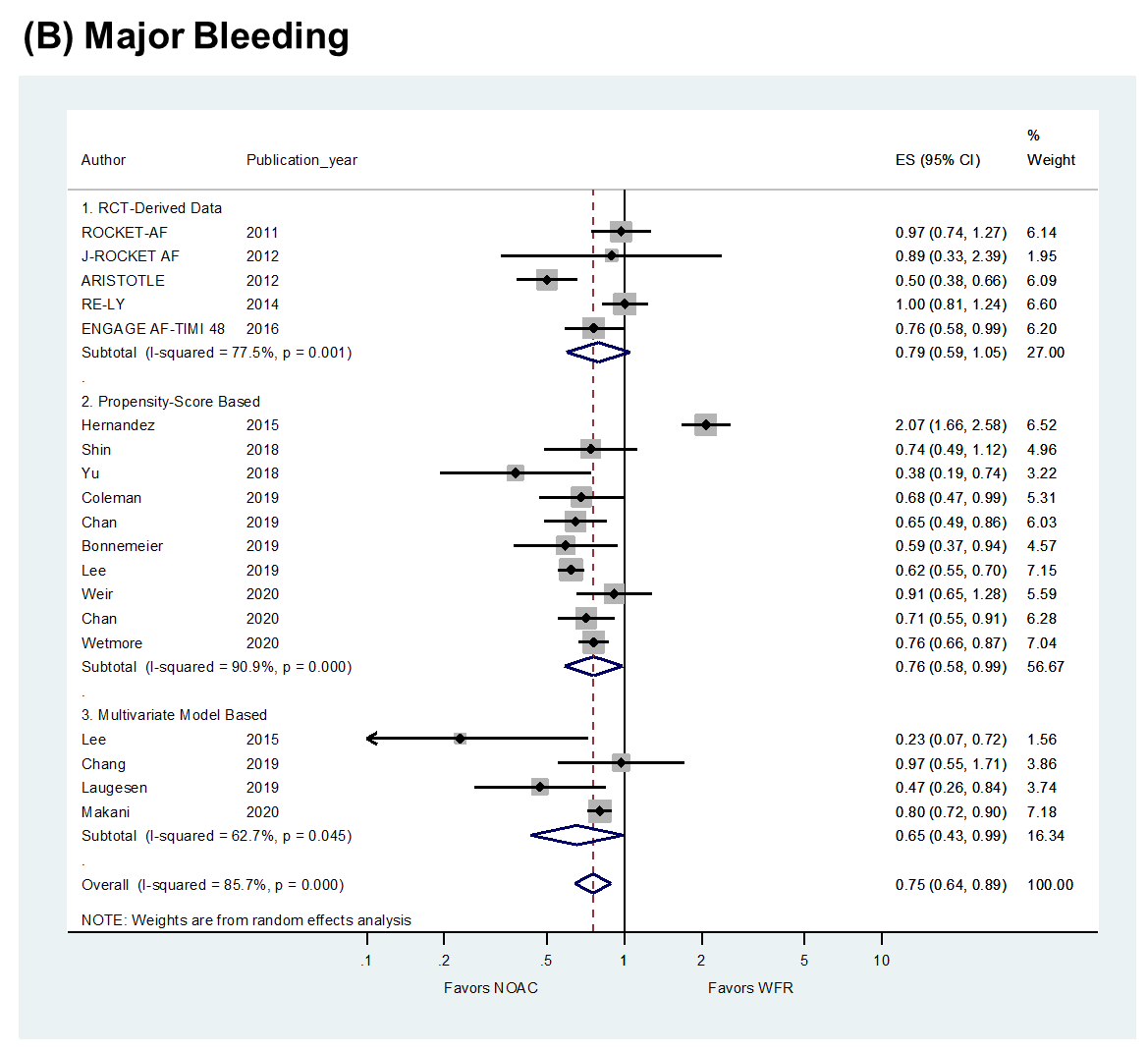
Supplementary Figure 6. Subgroup Analysis According to the Method of Adjustment for Baseline Differences**

**
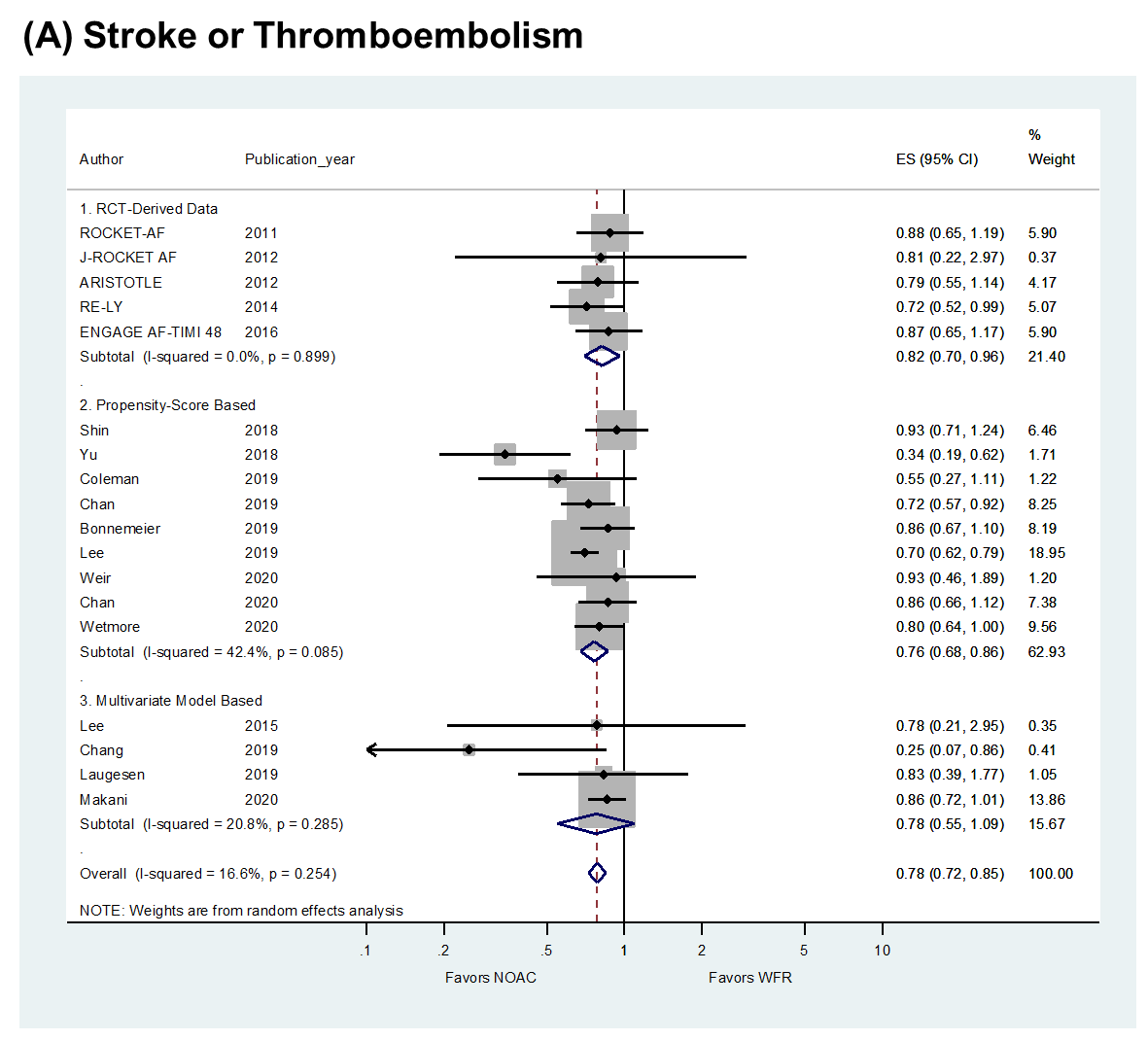
**

**
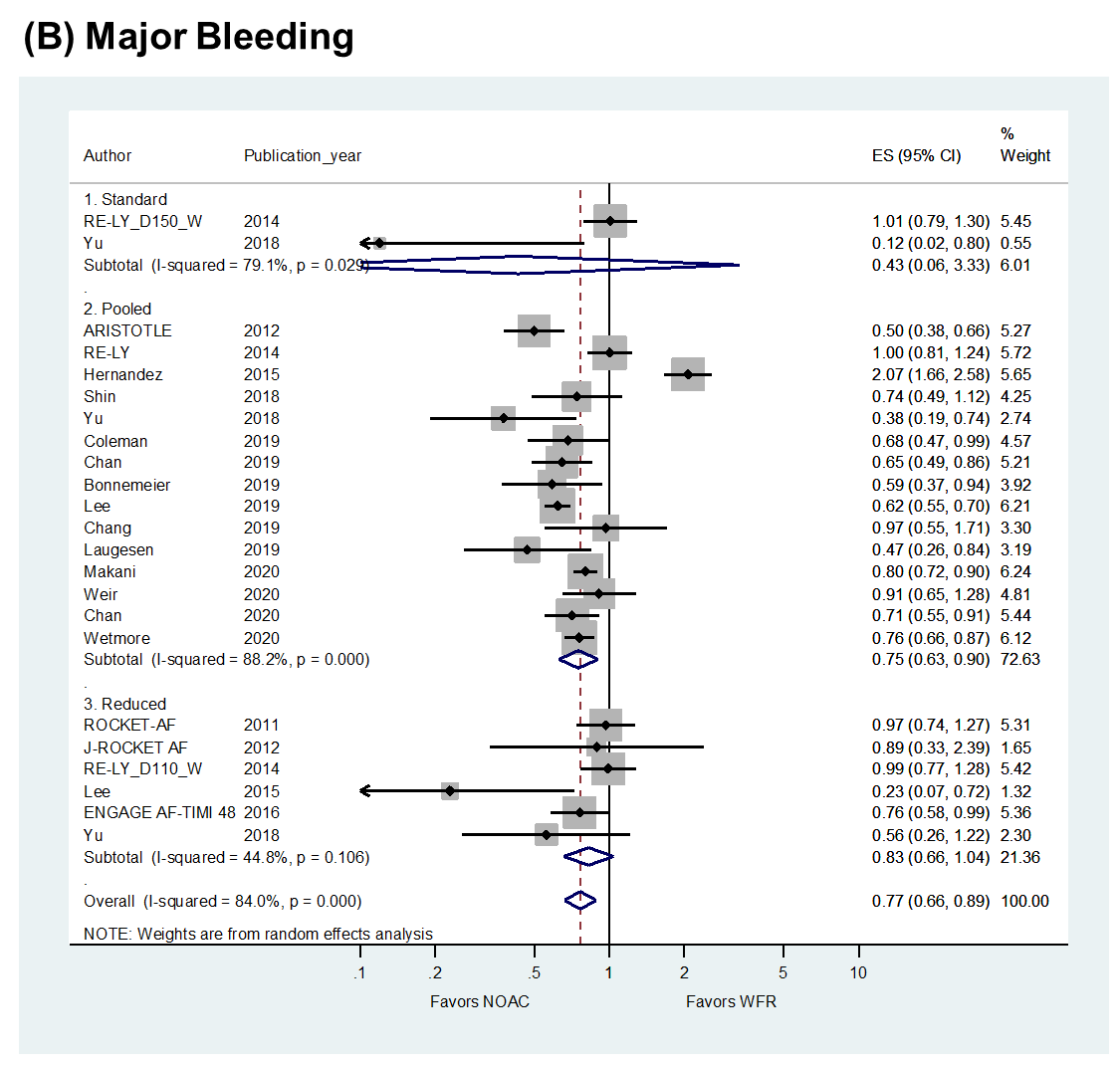
Supplementary Figure 7. Subgroup Analysis According to the Dose of Non-Vitamin K Oral Anticoagulants**

**
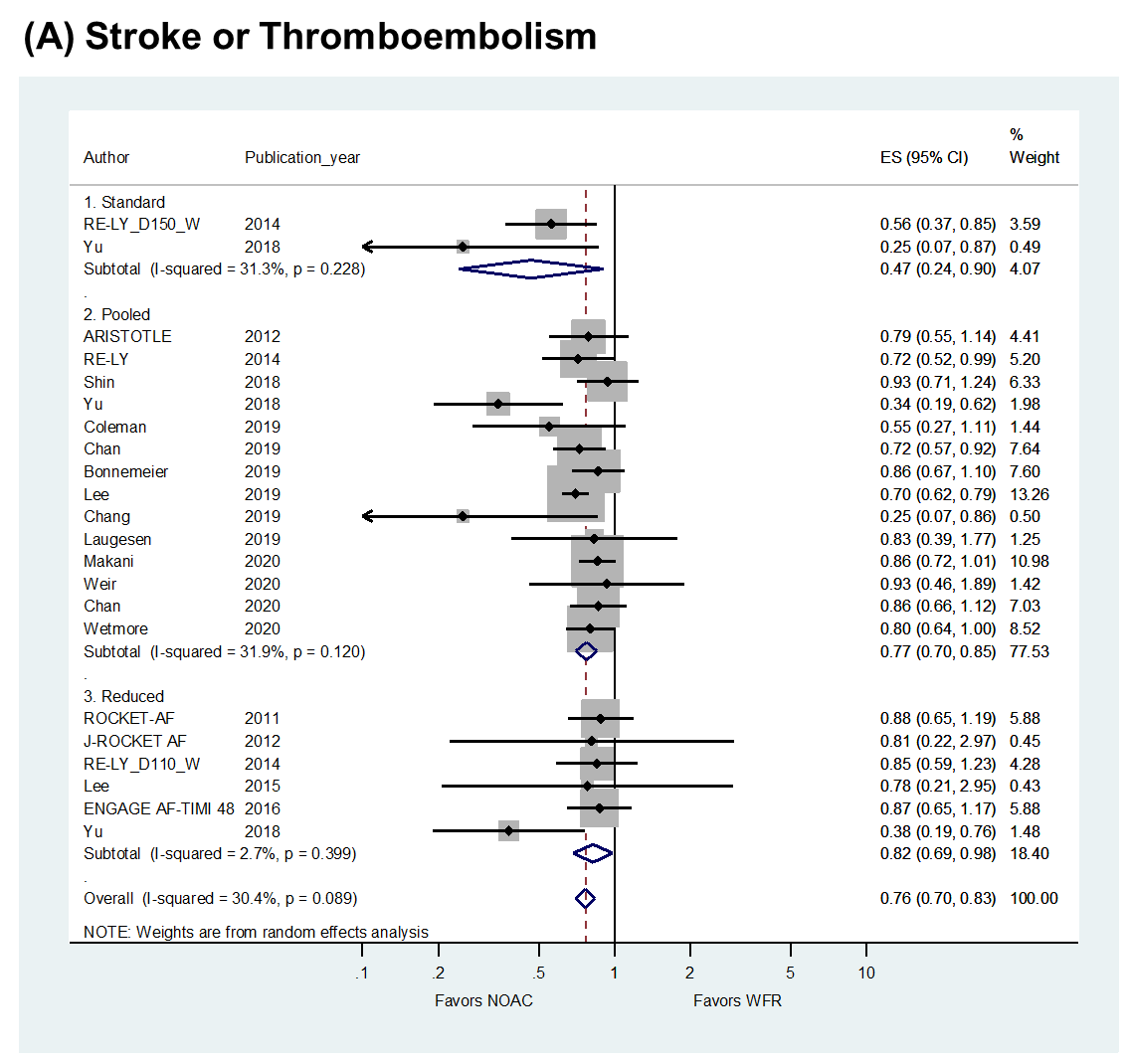
**

**
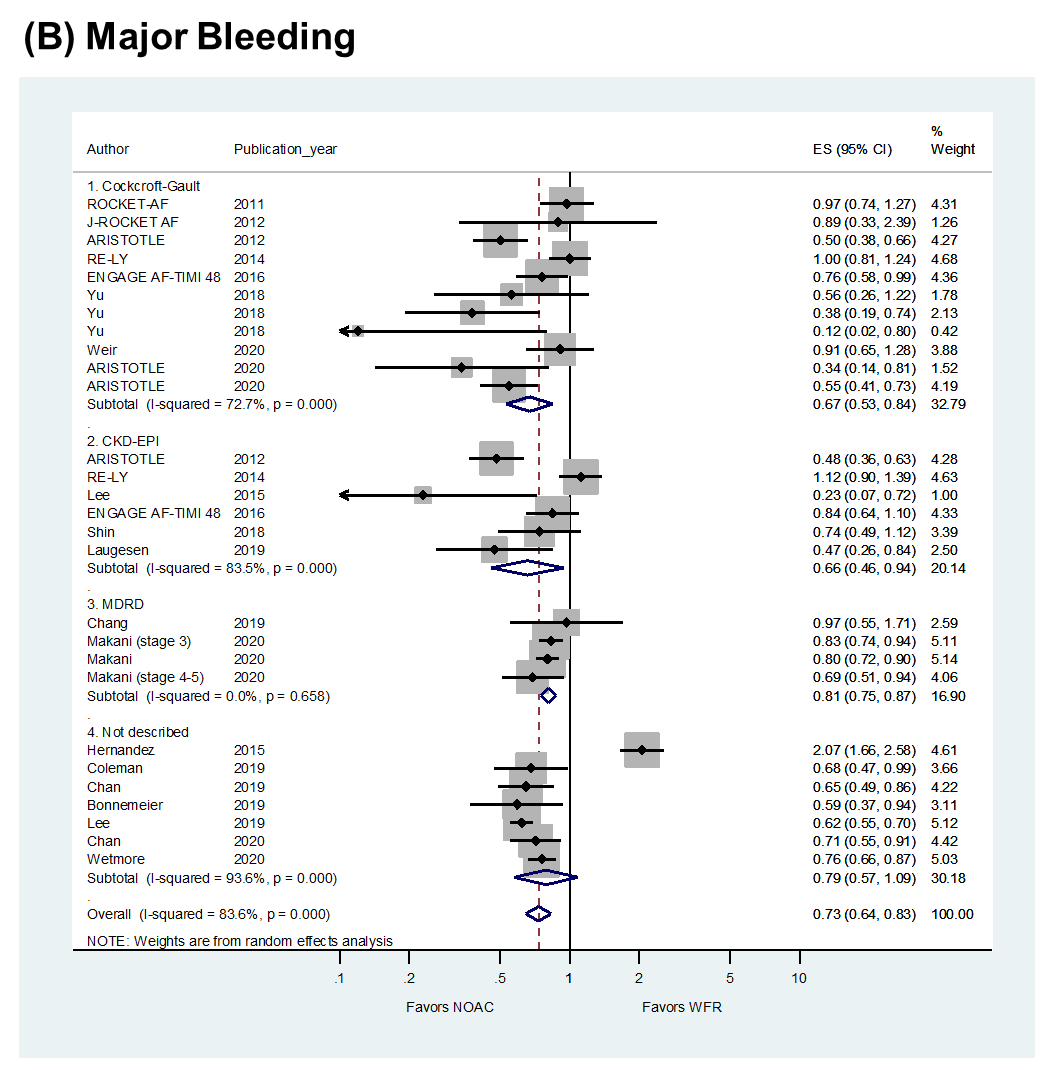
Supplementary Figure 8. Subgroup Analysis According to the Glomerular Filtration Rate Estimation Equation**

**
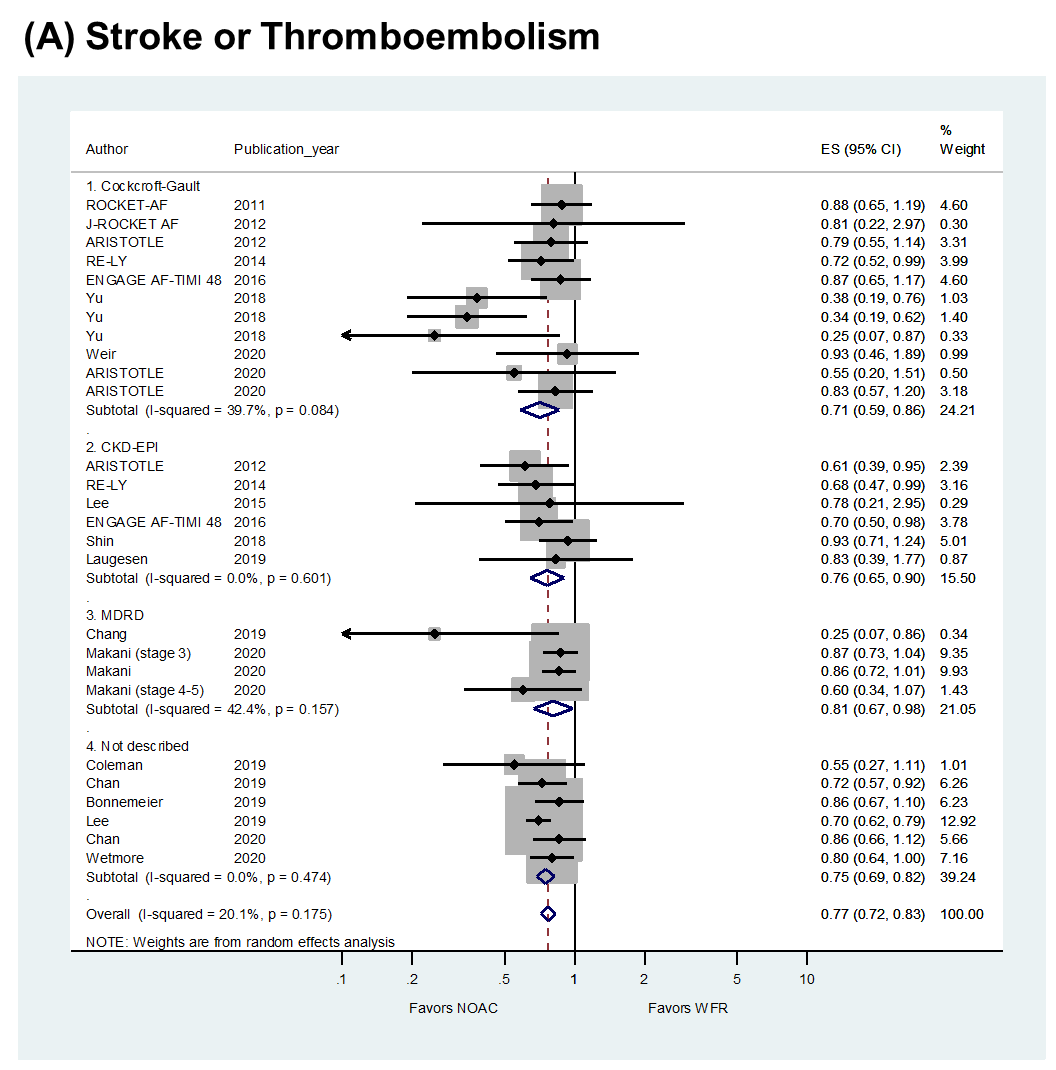
**

**Supplementary Figure 9. Validity of Frequentist Network Meta-Analysis for Stroke or Thromboembolism**

**
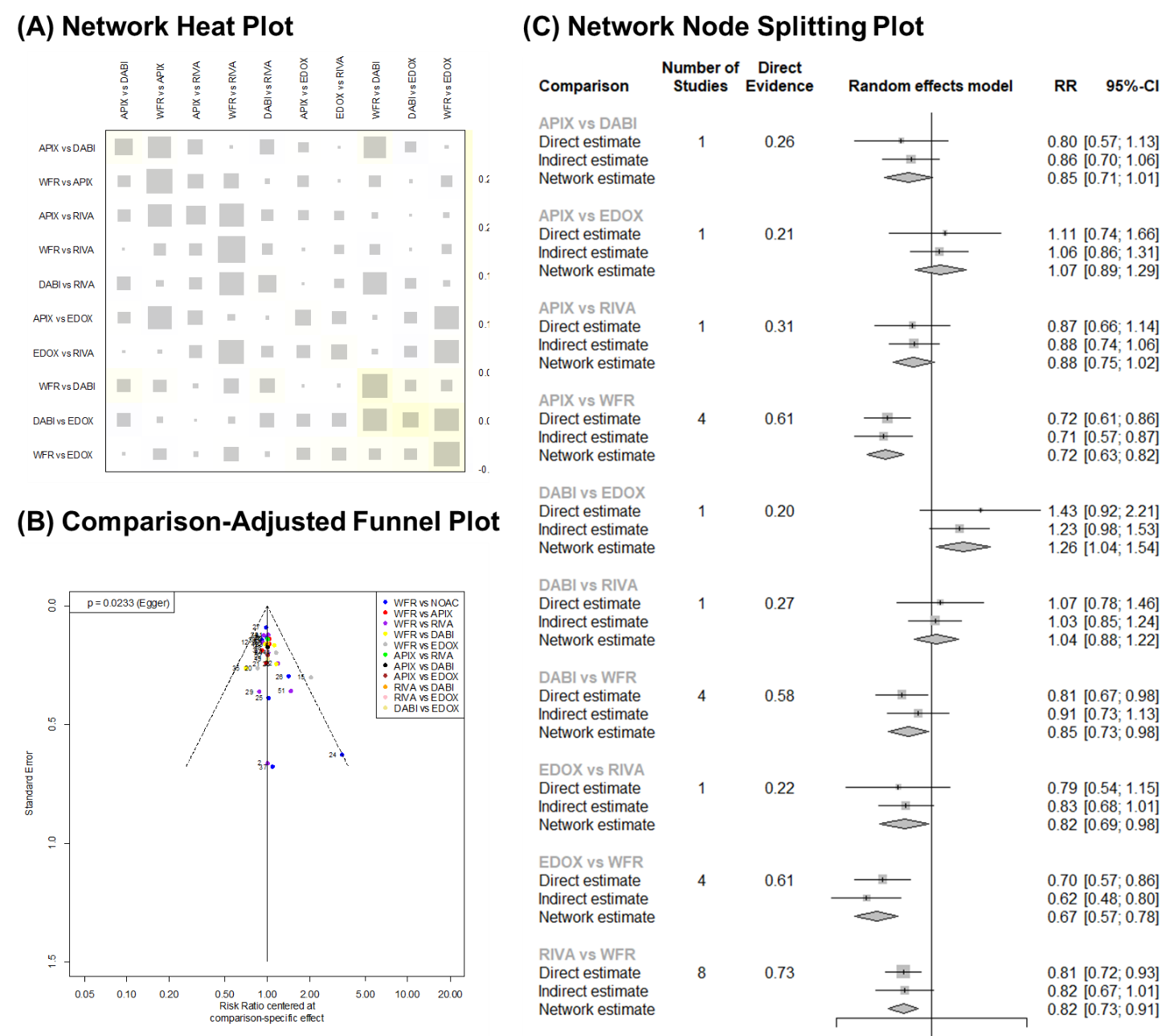
**

**Supplementary Figure 10. Validity of Frequentist Network Meta-Analysis for Major Bleeding**

**
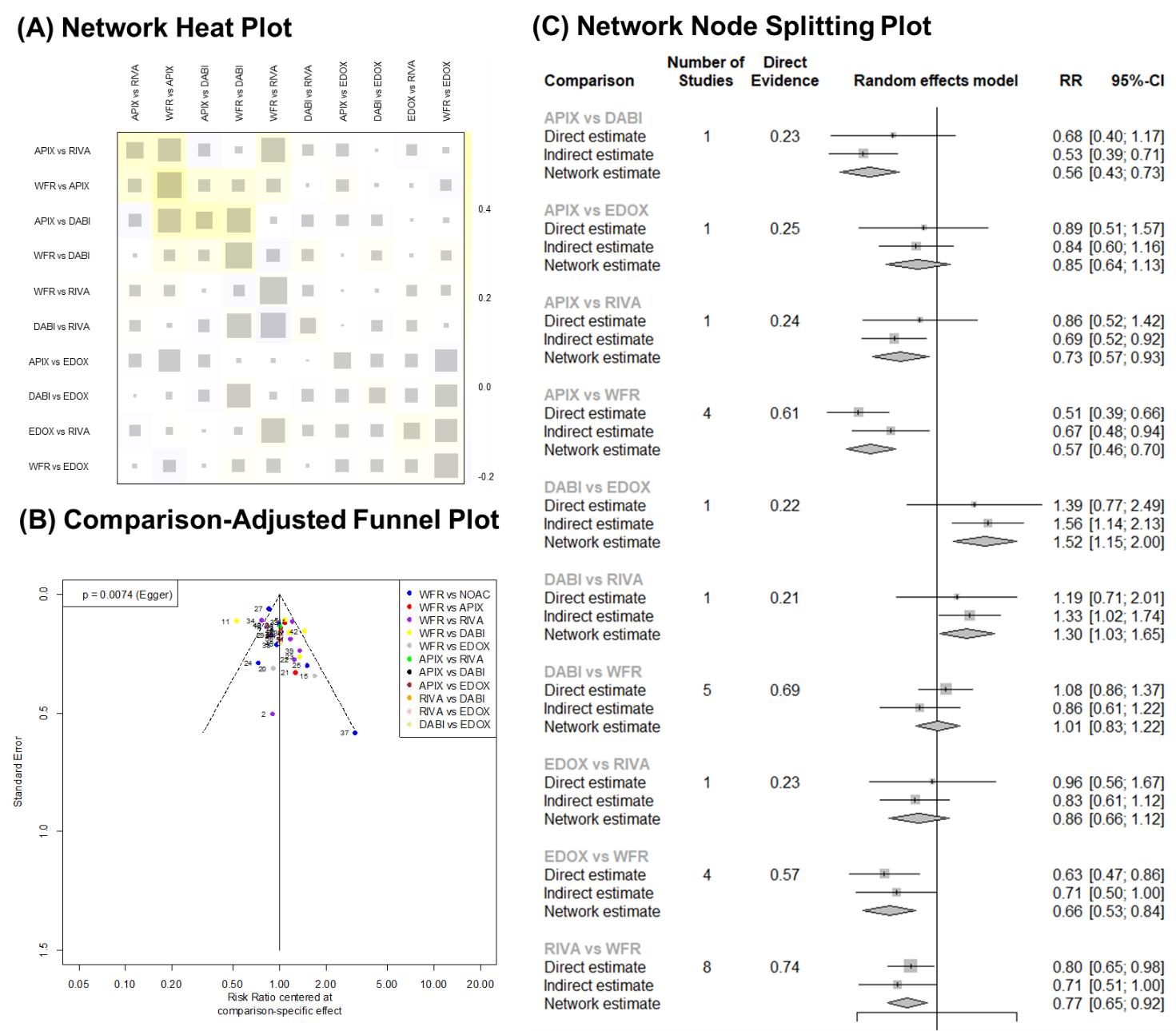
**
